# SALT: Introducing a framework for hierarchical segmentations in medical imaging using label trees

**DOI:** 10.1038/s41598-025-31639-1

**Published:** 2025-12-19

**Authors:** Sven S. Becker, Giulia Baldini, Cynthia S. Schmidt, Olivia B. Pollok, Obioma Pelka, Judith Kohnke, Katarzyna Borys, Christoph M. Friedrich, Benedikt M. Schaarschmidt, Michael Forsting, Lale Umutlu, Johannes Haubold, Felix Nensa, René Hosch

**Affiliations:** 1 Institute of Interventional and Diagnostic Radiology and Neuroradiology, University Hospital Essen, Essen, Germany; 2 Institute for Artificial Intelligence in Medicine (IKIM), University Hospital Essen, Essen, Germany; 3 Institute for Transfusion Medicine, University Hospital Essen, Essen, Germany; 4 Institute for Medical Informatics, Biometry and Epidemiology (IMIBE), University Hospital Essen, Essen, Germany; 5 Faculty of Computer Science, University of Applied Sciences and Arts, Dortmund, Germany

**Keywords:** Hierarchical segmentation, Deep learning, Medical imaging, Conditional probabilities, Efficient inference, Whole body imaging, Medical imaging

## Abstract

Traditional segmentation networks treat anatomical structures as isolated elements, often neglecting their hierarchical relationships. This study introduces Softmax for Arbitrary Label Trees (SALT), a novel method that leverages these hierarchical connections to improve segmentation efficiency and interpretability. SALT is a novel activation function that extends the softmax to arbitrary hierarchical label trees by modeling conditional probabilities along parent–child relations in imaging data. This enables anatomical hierarchies in CT imaging to be represented naturally, allowing segmentations to propagate from broader regions to detailed structures. Using the SAROS dataset from The Cancer Imaging Archive (TCIA), which comprises 900 body segmentations from 883 patients and was enhanced by TotalSegmentator to include 113 anatomical labels, the model was trained on 600 scans, with validation and testing on 150. Dice scores across SAROS, CT-ORG, FLARE22, LCTSC, LUNA16, and WORD datasets measured performance, with 95% confidence intervals (CI) from 1000 bootstrapping rounds. SALT achieved top performance on the LUNA16 and SAROS datasets, with Dice scores of 0.93 (95% CI: 0.919–0.938) and 0.929 (95% CI: 0.924–0.933). Reliable accuracy was also observed in CT-ORG (0.891, 95% CI: 0.869–0.906), FLARE22 (0.849, 95% CI: 0.844–0.854), LCTSC (0.908, 95% CI: 0.902–0.914), and WORD (0.844, 95% CI: 0.839–0.85). SALT can segment a 1000-slice CT in 35 s. By leveraging hierarchical body structures, SALT enables rapid whole-body segmentation, processing CT scans in 35 s on average. This capability supports its integration into clinical workflows, enhancing efficiency in automated full-body segmentation and contributing to improved diagnostic workflows and patient care.

**Supplementary Information:**

The online version contains supplementary material available at 10.1038/s41598-025-31639-1.

## Introduction

Computed Tomography (CT) imaging stands out as one of the most comprehensive tools in the field of diagnostic imaging, with the number of CT scans increasing by 4% per year worldwide^1^. With the rising volume of CT scans, radiologists face a growing workload, underscoring the need for automated solutions to support them. Moreover, CT scans contain a substantial volume of information, and only a portion is used for specific diagnosis purposes. A considerable amount of potentially clinically valuable information remains unexplored and is the focus of current research^2^. In this context, deep learning networks can automate tasks and extract information from scans with minimal additional cost, aside from algorithm training. In particular, automated segmentation of CT scans is now a widespread technique for identifying key anatomical features such as organs, tissues, and vessels^2,3^. These detailed segmentations aid radiologists in making accurate diagnoses and have been linked to indicators of a patient’s well-being^4^.

Furthermore, CT scans enable the automated calculation of Body Composition Analysis (BCA), which quantifies the amount of fat, muscle, and bone^5^ and is proving to be valuable in different clinical endpoints ^6–8^. The currently existing models for full-body segmentation, such as TotalSegmentator^2^, often rely on multiple models for segmenting CT scans when the number of labels becomes too extensive for a single model to handle efficiently. However, completing a full segmentation- typically still requires around 5–10 min per CT scan on a modern GPU^9^, which is impractical for scenarios where segmentation algorithms are expected to operate continuously as part of a hospital’s automation or preprocessing pipelines. In response, we introduce the Softmax for Arbitrary Label Trees (SALT) framework, an approach that employs a single, robust model to manage a broad spectrum of labels efficiently. The SALT framework harnesses hierarchical relationships to segment a vast range of anatomical landmarks, reflecting the natural tree-like organization of the human anatomy. By employing conditional probabilities, this framework models the intricate relationships between these landmarks, capturing the complex network of connections among various anatomical structures. In this study, an application of the SALT framework to 3-dimensional CT scans using a nnUNet^9^ architecture is presented. However, the flexibility of this framework allows for its adaptation to other contexts, accommodating both 2D and 3D imaging across diverse image types, and it could be used with a wide range of deep learning algorithms. This adaptability underscores the SALT framework’s potential as a universal tool for medical image analysis. This optimization aims to resolve existing bottlenecks and substantially improve the utility of CT scan data in real-time clinical settings, facilitating the seamless operation of the segmentation algorithm within the hospital’s data acquisition process.

## Materials and methods

### Datasets

**Fig. 1:**
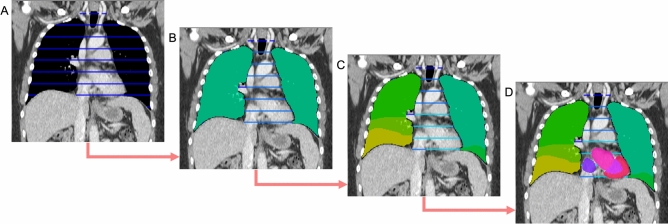
Example of a hierarchical segmentation. (**A**) In the thorax, the biggest region is the thoracic cavity. (**B**) The thoracic cavity can then be subdivided into lungs and mediastinum. (**C**) The mediastinum has a further subregion the pericardium, and the lungs can be subdivided into lower/middle/upper left/right lobes. (**D**) The pericardium contains the heart, which can be subdivided into myocardium and left/right ventricle/atrium. The full segmentations originate from the TotalSegmentator, while the sparse segmentations are from SAROS.

This study used a selection of datasets available on The Cancer Imaging Archive (TCIA)^10^ to train and evaluate the SALT approach. For the training, 750 CT scans from the Sparsely Annotated Region and Organ Segmentation (SAROS)^11^ dataset were used (600 for training and 150 for validation). In this dataset, the segmentations target anatomical landmarks that are relevant for body composition analysis (BCA)^5,12^. The annotations cover a wide range of areas such as the abdominal and thoracic cavities, bones, brain, mediastinum, muscles, pericardium, spinal cord, and subcutaneous tissue. In addition to these annotations, segmentations of organs, vessels, and specific muscles and bones were generated using Version 1 of the TotalSegmentator models^2,13^ for the same dataset of 750 scans. The TotalSegmentator predictions were then fused with the SAROS annotations to create a single dataset of 750 scans containing all labels. For SAROS, smaller labels such as the thyroid, submandibular, and parotid glands were not included in the final segmentation. This exclusion was not due to any limitation of the SALT method but rather because these labels were sparsely represented in SAROS and more consistently available from TotalSegmentator, which already provides high-quality gland segmentations. Thus, the merged dataset prioritizes label consistency and coverage across larger and smaller anatomical structures. This fusion is illustrated in Fig. 1, which highlights the natural tree-like organization of the human body. For example, the body encompasses the thoracic cavity, which itself includes organs like the lungs and heart. These organs, in turn, can be subdivided further into more specific segments, such as the lobes of the lungs and the atria and ventricles of the heart.

For the evaluation, an additional independent test set of 150 CT scans from SAROS and other publicly available datasets were used: CT Volumes with Multiple Organ Segmentations (CT-ORG)^14^, Fast and Low-resource Semi-supervised Abdominal Organ Segmentation (FLARE22)^15,16^, Lung CT Segmentation Challenge (LCTSC)^17,18^, Lung Nodule Analysis 2016 (LUNA16)^19^, and Whole Abdominal Organ Dataset (WORD)^20^. These datasets primarily cover thoracic and abdominal regions, as these were the areas consistently annotated and publicly available across sources. Consequently, head, neck, and pelvic organs were not included in the current evaluation; notably, the SALT framework itself is not limited to these regions and can be applied to whole-body segmentation if sufficient annotated data are available. The “gallbladder” label from the WORD dataset was omitted because the masks were either very few points and not an actual segmentation, a segmentation of the common bile duct, or segmentations of tumors (see Supplementary Figure S1). The final composition of the evaluation dataset, together with the number of cases and the labels used, is reported in Table 1.

**Table 1:** Datasets used for the evaluation.

Dataset	Number of Scans	Labels
CT-ORG^10,14,21^	21	Liver, bladder, lungs, kidneys, bone, brain
FLARE22^15,16^	50	Liver, right kidney, spleen, pancreas, aorta, inferior vena cava, right adrenal gland, left adrenal gland, gallbladder, stomach, duodenum, left kidney
LCTSC^10,17,18^	60	Spinal cord, lung right, lung left, heart
LUNA16^19,22^	51	Upper right lobe, middle right lobe, lower right lobe, upper left lobe, lower left lobe
SAROS^10,11^	150	Subcutaneous tissue, muscles, abdominal cavity, thoracic cavity, bones, mediastinum, pericardium, brain, spinal cord
WORD^20^	140	Liver, spleen, kidney left, kidney right, stomach, pancreas, duodenum, colon, intestine, adrenal glands, bladder

Multiple datasets from TCIA were used to evaluate SALT: CT Volumes with Multiple Organ Segmentations (CT-ORG), Fast and Low-resource Semi-supervised Abdominal Organ Segmentation (FLARE22), Lung CT Segmentation Challenge (LCTSC), Lung Nodule Analysis 2016 (LUNA16), Sparsely Annotated Region and Organ Segmentation (SAROS), and Whole Abdominal Organ Dataset (WORD). These datasets primarily include thoracic and abdominal organs, which were consistently annotated across public datasets; head, neck, and pelvic organs were not part of the current evaluation. SALT, however, is not restricted to these regions and can generalize to full-body segmentation given sufficient annotated data. For SAROS, an independent test set was used for the evaluation. For each dataset, the number of included CT scans and the labels used for assessment are reported.

### Dataset postprocessing

The labels from all datasets underwent postprocessing to ensure the hierarchical structure by either merging or splitting the original segmentations. An example of merging is the lung label, which was not present in the TotalSegmentator labels but could be inferred by combining the upper, lower, middle left, and right lobes. In some cases, splitting was necessary to ensure the tree structure, as each label can only have one parent. For instance, the aorta passes through the mediastinum, pericardium, and abdominal cavity, which would imply three parents. To preserve the tree structure, the aorta label was split into three separate regions: “aorta thoracica pass pericardium”, "aorta thoracica pass mediastinum", and “aorta abdominalis”. Similar splitting was performed for the inferior vena cava and pulmonary artery.

**Fig. 2:**
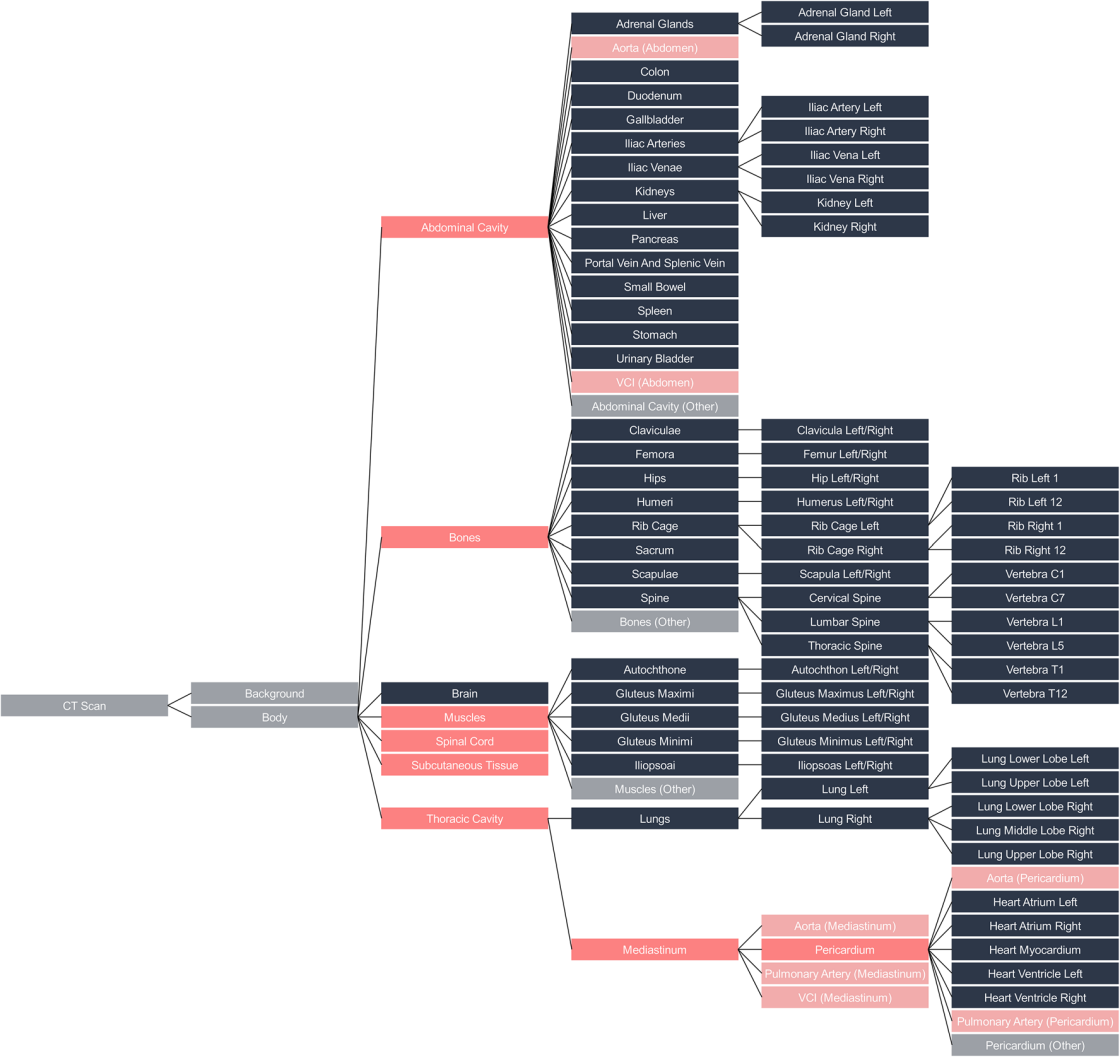
Hierarchical labeling for the segmentation of anatomical landmarks. The blue labels were generated with the TotalSegmentator, while the pink labels come from the SAROS dataset. The gray labels were also generated: “body” is the sum of all annotated voxels, while “background” is all non-annotated voxels. The “other” classes were created as the parts of the parents which were not annotated. The light pink labels were generated by splitting an existing TotalSegmentator label. The complete list of all vertebrae and ribs have been removed from this visualization for a better overview. For some bones and muscles with only left and right children, the two nodes were fused for better visualization.

Furthermore, in cases where the child labels were insufficient to annotate the volume of the parent label fully, additional labels were created. For instance, the thoracic cavity has two child labels, namely the lungs and the mediastinum (as depicted in Fig. 1). However, these two volumes alone do not encompass the entire thoracic cavity. Therefore, an additional label (called “other”) was introduced to incorporate the remaining voxels and ensure a correct learning process for the model. In addition, the SAROS dataset includes sparse annotations, with only every fifth slice reviewed by a medical expert. To obtain dense and volumetric segmentations for BCA, we used the validated Body and Organ Analysis (BOA) model, which provides reliable full-volume predictions for BCA-related structures. In addition, TotalSegmentator was applied to generate a broader set of anatomical labels beyond the BCA regions. After postprocessing, there were a total of 145 labels (excluding the root) and 113 anatomical leaf nodes. The hierarchical structure of the labels can be viewed in Fig. 2 and the detailed label distribution is presented in Supplement Tables S9 and S10.

While this approach provided anatomically consistent segmentations across organs, we acknowledge that the resulting labels do not represent a manually curated ground truth. A subset of the generated annotations was reviewed by a board-certified radiologist (7 years of experience), and no systematic errors or extreme outliers were identified. However, due to limited resources, a complete manual validation was not performed. This limitation is reflected in the Failure Analysis section, where the potential impact of residual annotation inconsistencies on model performance is discussed.

### Implementation details softmax for arbitrary label trees

**Fig. 3:**
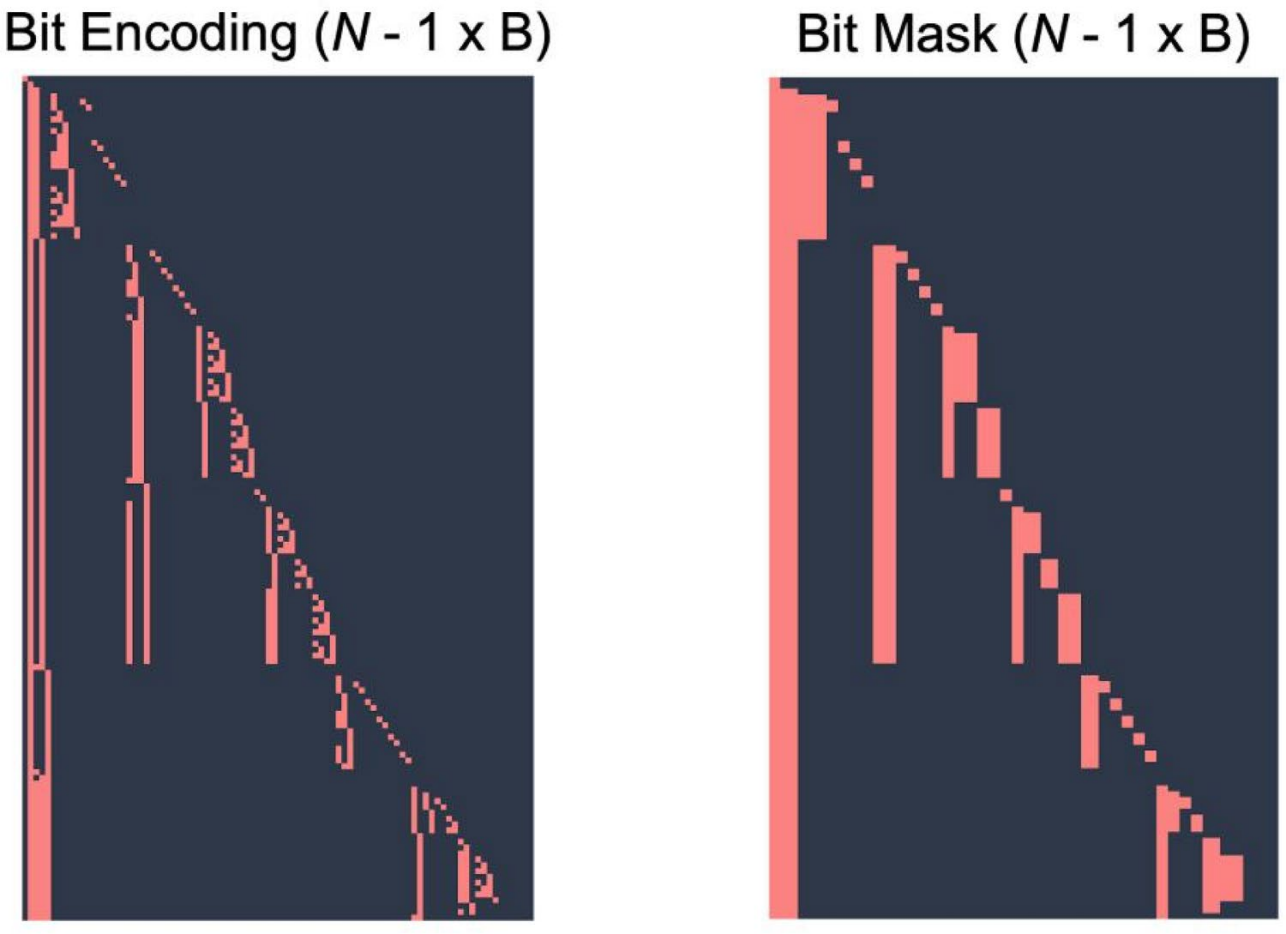
Bitwise representation of the class hierarchy. Both the bitwise encoding and the bitwise mask have size *NXB* , representing the number of classes (*N*) and the number of bytes (*B*) needed to identify each node uniquely.

The name SALT reflects the core concept of our method: a generalization of the standard softmax activation to operate over arbitrary hierarchical label trees. By modelling conditional probabilities along parent–child relationships, SALT produces anatomically consistent predictions across hierarchical levels, enabling flexible and efficient segmentation of complex label structures. To enable anatomically consistent segmentation and evaluation within hierarchical label structures, SALT integrates three key components: hierarchical encoding for evaluation, hierarchical supervision via reachability matrices, and conditional probability modeling. This integration forms a unified approach that enforces semantic dependencies throughout both training and inference. Standard segmentation metrics and losses treat each class independently, ignoring parent–child relations within anatomical ontologies. We therefore represent the class hierarchy as a tree in which each node receives a compact bitwise encoding describing its ancestral path from the root (see Fig. 3).

**Fig. 4:**
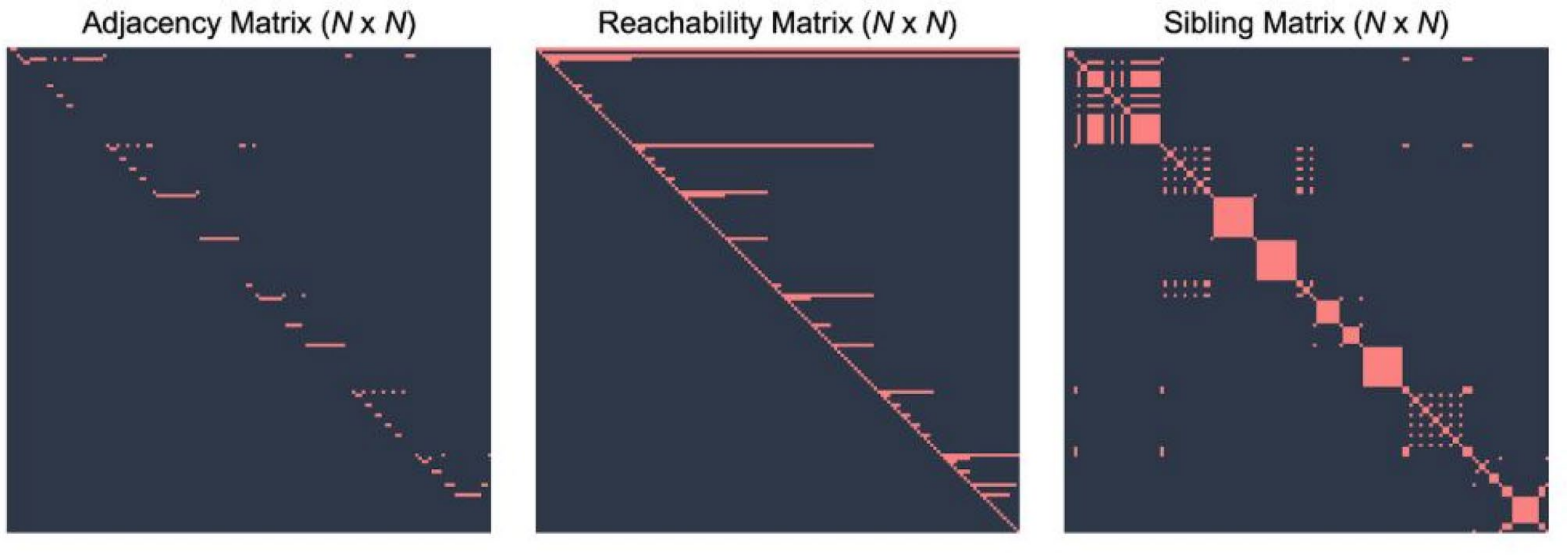
Different representations of the tree class hierarchy. From left to right: (1) The adjacency matrix encodes directed edges from parent to child nodes. (2) The reachability matrix encodes all nodes in the path from the root node to a specific node. (3) The sibling matrix encodes all sibling nodes for a specific node.

This allows voxel-level comparisons to be performed in the encoded space, eliminating the need to merge or flatten masks. The resulting *hierarchical Dice* extends the conventional Dice coefficient by incorporating these encodings, enabling performance evaluation that reflects structural similarity across levels of the hierarchy. Details of the encoding scheme and voxel-wise bit operations are provided in Supplementary Text T1**.** During training, we propagate supervision signals through the hierarchy using an adjacency and a reachability matrix that encode parent–child and ancestor relations between classes. Each voxel label is mapped to its corresponding reachability vector, which activates not only the target node but also all of its ancestors. This representation ensures that predictions of fine-grained classes are conditioned on the correct identification of their higher-level structures, linking Dice and cross-entropy optimization across multiple semantic levels (see Fig. 4). Matrix definitions and construction procedures are detailed in Supplementary Text T2**.**

The SALT activation layer generalizes the softmax function to arbitrary label trees. For each node, the network predicts a conditional probability that is normalized over its sibling group via a softmax operation. The final probability for any node is obtained by chaining these conditional probabilities along the path from the root to that node. This enforces hierarchical normalization and guarantees that no child node can receive a nonzero probability unless its ancestors are also active. It thus integrates anatomical dependencies directly into the network’s probabilistic output space. The derivation and implementation of the SALT layer are described in Supplementary Text T3.

### Model preprocessing and training

**Fig. 5:**
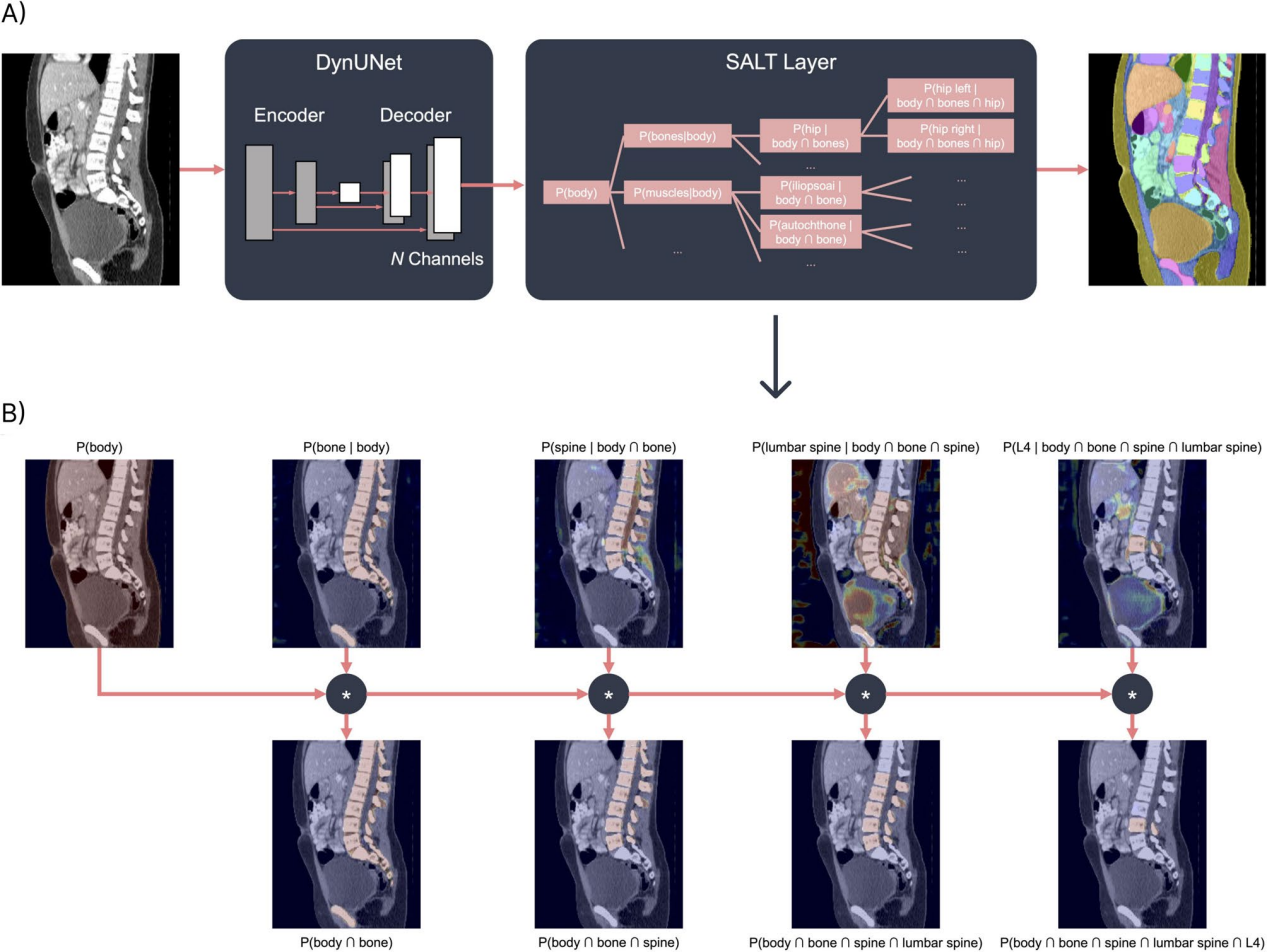
Overview of the SALT architecture and process. (**A**) A DynUNet model outputs feature maps with *N* channels, where *N* corresponds to the number of nodes in the hierarchical label tree. The SALT layer then computes conditional probabilities along parent–child paths and replaces the standard softmax for generating structured segmentations. (**B**) Example visualization of the conditional probability maps used to segment the L4 vertebra. The model does not predict the target class directly but infers it through the hierarchical path: spine → lumbar spine (LS) → L4.

The SALT architecture in this paper consists of a DynUNet from the MONAI framework^23^ (version 1.1, PyTorch^24^ version 1.14), which is a reimplementation of the architecture utilized by nnUNet^9^. The output feature map of the DynUNet model has 145 channels, corresponding to all nodes in the hierarchical label tree used for whole-body segmentation. Across these channels, the SALT activation function is applied as the final layer. SALT implements a conditional softmax that operates along each parent–child path in the hierarchical tree, ensuring that the predicted probability of each child node is consistent with its ancestor nodes. This structure enables SALT to enforce anatomical consistency across segmentation outputs while leveraging the complete label hierarchy. Figure 5 illustrates this process.

Initially, all CT scans underwent several pre-processing steps: Left-Posterior-Inferior voxel reorientation, resampling to a voxel spacing of 1.5 × 1.5x1.5 mm, normalization of intensity values within the Hounsfield Units to scale the range from -1024 to 1024 to between 0 and 1, and random crops of size 192 × 192x48. The model was trained for 1000 epochs with 600 CT scans for training and 150 for validation. The AdamW optimizer^25^ was used with an initial learning rate of 0.00025 and a weight decay of 0.00005. The learning rate was reduced during training using a cosine function. The model used a hybrid loss based on categorical cross-entropy loss and Dice loss, which are commonly used for medical segmentation tasks^9^. In contrast to the common use of these losses, SALT optimizes multiple classes simultaneously by constructing an encoding of the nodes using the reachability matrix. For the calculation of the evaluation metrics during training, a similar approach that uses the tree structure to encode each node was used. Furthermore, the trained model and the code are available for review on GitHub under the following link: https://github.com/UMEssen/SALT.

### Evaluation

The model’s performance was evaluated using the Dice score^26^ and normalized surface Dice score (NSD)^27^, with NSD measuring the frequency of surface distances under 3 mm, as used by TotalSegmentator. For comparison, Version 2 of TotalSegmentator^2,9^ was also assessed on datasets from Table 1 using these metrics. Confidence intervals (95%) were calculated via 1000 bootstrapping iterations, using the 2.5 and 97.5 percentiles as bounds. Dataset score distributions for Dice and NSD were compared for shared organ labels. Model speed was evaluated against TotalSegmentator Versions 1 and 2 across multiple spacing settings (1.5 mm, 3 mm, and 6 mm), all tested on a single NVIDIA RTX A6000 card.

## Results

### Segmentation evaluation

The trained model showed a Dice of 0.891 [0.887, 0.896] and an NSD of 0.931 [0.927, 0.936]. An overall score for the different datasets is presented in Table 2 together with the scores obtained by Version 2 of the TotalSegmentator. The Dice scores and the NSD scores for the datasets are reported in Supplementary Table S1, Supplementary Table S2, Supplementary Table S3, Supplementary Table S4, Supplementary Table S5, and Supplementary Table S6. Notably, the lungs, the liver, the spleen, and the stomach achieved the best scores across the different datasets.

**Table 2:** Summary results of the model’s performance for the datasets.

Metric	CT-ORG	FLARE22	LCTSC	LUNA16	SAROS	WORD
SALT (Dice)	0.891 [0.869, 0.906]	0.849 [0.844, 0.854]	0.908 [0.902, 0.914]	0.93 [0.919, 0.938]	0.929 [0.924, 0.933]	0.844 [0.839, 0.85]
TSV2 (Dice)	0.917 [0.898, 0.931]	0.892 [0.888, 0.897]	0.937 [0.931, 0.942]	0.952 [0.943, 0.959]	0.852 [0.846, 0.857]	0.84 [0.834, 0.846]
SALT (NSD)	0.884 [0.863, 0.905]	0.947 [0.942, 0.952]	0.886 [0.872, 0.899]	0.908 [0.89, 0.92]	0.98 [0.975, 0.984]	0.909 [0.903, 0.915]
TSV2 (NSD)	0.916 [0.895, 0.933]	0.954 [0.949, 0.959]	0.945 [0.935, 0.954]	0.934 [0.925, 0.943]	0.955 [0.949, 0.959]	0.906 [0.899, 0.912]

The evaluation is performed in terms of Dice and Normalized Surface Dice (NSD) for both SALT and Version 2 of the TotalSegmentator (TSV2). The 95% confidence intervals were computed using 1000 rounds of bootstrapping, and the interval is reported in square brackets.

**Fig. 6:**
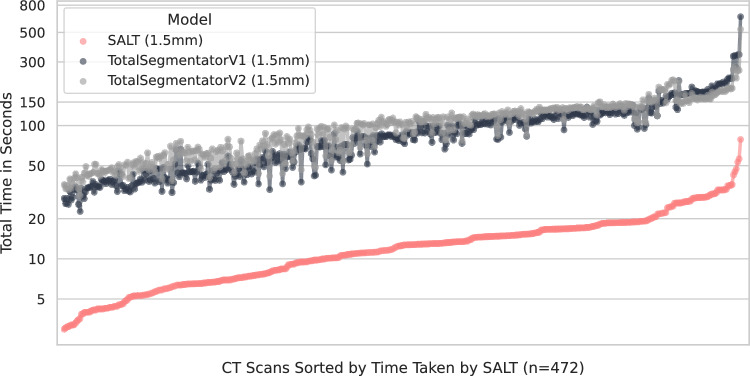
Comparison between the speed of SALT and the TotalSegmentator. Version 1 and 2 of the TotalSegmentator were run on the same set of 472 CT scans from Table 1. The TotalSegmentator models and SALT were trained on 1.5 mm isotropic spacing.

Additionally, an evaluation of the speed of the model at inference time was also performed, which can be reviewed in Supplementary Table S7. It is relevant to make a distinction between the inference time (the time the model takes to make a prediction) and the total time, as a large portion of the inference time is just spent postprocessing and storing the result. In Fig. 6, a comparative analysis of SALT’s speed against both Version 1 and Version 2 of the TotalSegmentator is presented. This comparison clearly demonstrates that SALT consistently outperforms its counterparts, showing a notable speed advantage, especially with larger CT scans. Additionally, a speed comparison was conducted between SALT and the faster variants of TotalSegmentator that utilize lower spacing, with the findings detailed in Supplementary Figure S2.

These “fast” alternatives of TotalSegmentator, which use a single model instead of five, also exhibit better speed. In these comparisons, the 3 mm model from Version 2 of TotalSegmentator was consistently slower than or comparable to SALT. However, for larger images, the 3 mm model of Version 1 and the 6 mm model of Version 2 were faster.

### Failure analysis

**Fig. 7:**
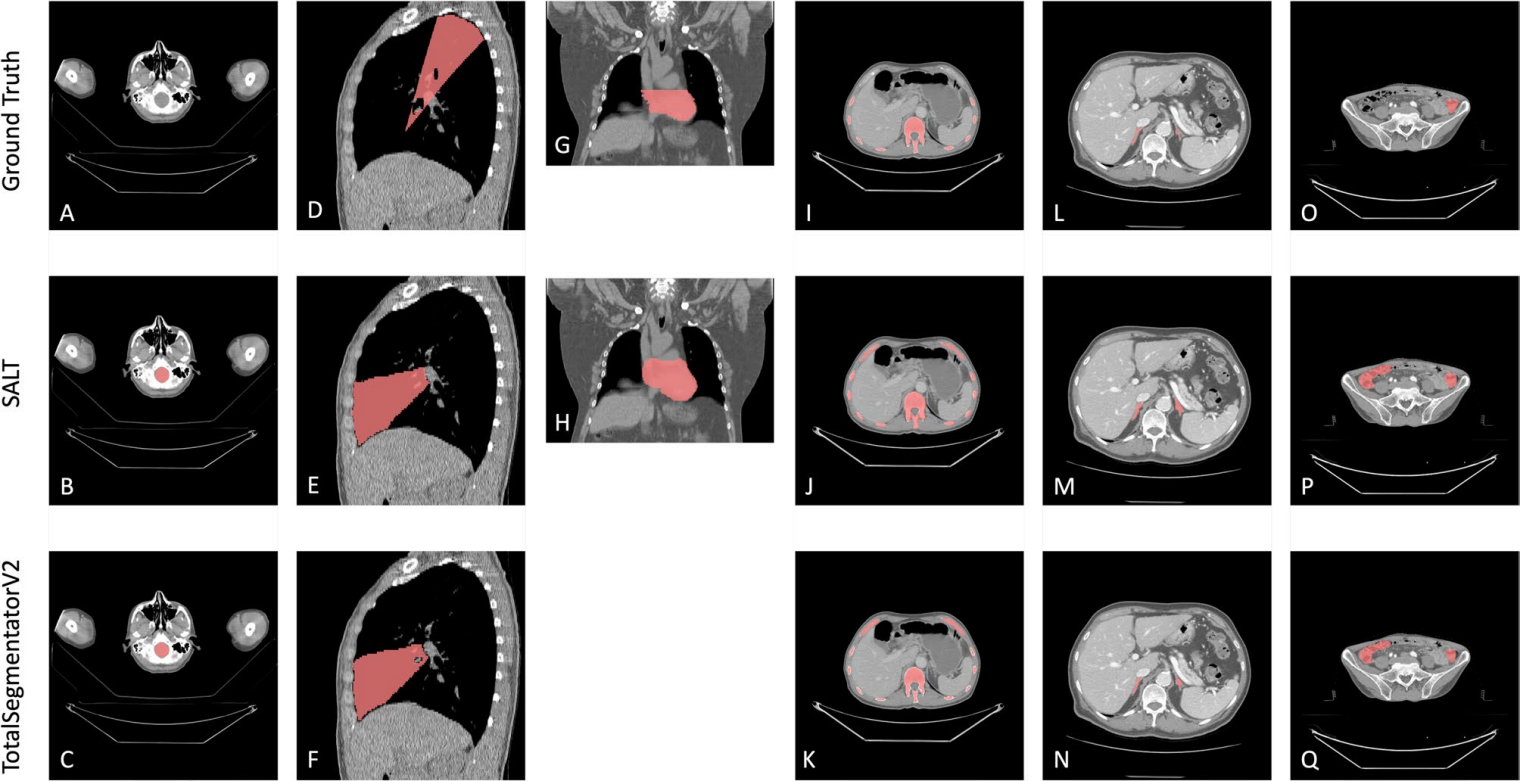
Comparison between the ground truth (first row), SALT (second row) and Version 2 of the TotalSegmentator (third row). The comparisons across various classes and datasets are systematically organized as follows: Figures (**A**), (**B**), and (**C**) focus on the comparison of the brain class from the CT-ORG dataset. Figures (**D**), (**E**), and (**F**) examine the middle lobe of the LUNA16 dataset. In Figures (**G**) and (**H**), the analysis shifts to the pericardium from the LCTSC dataset. The bone class from the CT-ORG dataset is compared in Figures (**I**), (**J**), and (**K**). Figures (**L**), (**M**), and (**N**) represent the adrenal glands from the FLARE22 datasets. Finally, Figures (**O**), (**P**), and (**Q**) compare the colon class from the WORD dataset.

The model’s performance exhibited notable variation across datasets for specific classes (Fig. 7), as evidenced by statistically significant discrepancies in shared labels (Supplementary Table S8). In the case of the brain class, SAROS achieved a Dice score of 0.758, while CT-ORG attained a significantly lower score of 0.486. This discrepancy was attributed to inconsistencies in the annotation process. In the CT-ORG dataset, only a single scan was annotated for the brain, despite the presence of visible brainstem segments in another scan that were classified by SALT but not labeled in the dataset. TotalSegmentator produced comparable results, reflecting the challenges associated with handling partial brain segments (Figs. 7B-C).

The LUNA16 dataset demonstrated generally accurate lung segmentations, although the right middle lobe presented particular challenges. Both SALT and TotalSegmentator exhibited occasional misclassification of this region, resulting in a Dice score of 0.848 (see Supplementary Table S4). This highlights the challenges associated with segmenting lung regions with indistinct boundaries (Figs. 7E-F). The application of different annotation guidelines resulted in Dice scores of 0.894 for LCTSC and 0.952 for SAROS for the pericardium. There was a discrepancy between the annotations provided by LCTSC and those produced by SALT. LCTSC had annotated the heart around the pericardial sac, whereas SALT had segmented the entire area within the sac. This resulted in a mismatch (Fig. 7H). Furthermore, discrepancies were observed in the bone class; SALT achieved Dice scores of 0.911 in SAROS and 0.872 in CT-ORG. It should be noted that the SALT segmentation included cortical bone, which was not present in the CT-ORG or TotalSegmentator segmentations. Furthermore, SAROS annotated rib cartilage as bone, which differs from the approach taken by CT-ORG (Figs. 7I-K). The adrenal glands exhibited the lowest Dice scores (0.65–0.706), which can be attributed to SALT’s proclivity to incorporate surrounding tissue. Notwithstanding the discrepancies above, the high NSD scores (0.887–0.957) indicated accurate boundary alignment (Figs. 7L-N). Colon segmentation also presented difficulties, with occasional misidentification of air-filled small bowel sections as colon, particularly in cases of right hemicolectomy. This misclassification was observed in both SALT and TotalSegmentator, with Dice scores of 0.808 for SALT and 0.773 for TotalSegmentator (Figs. 7O-Q). These examples demonstrate the impact of anatomical variability and inconsistent annotation practices across datasets.

## Discussion

In this work, we propose the SALT framework to provide a single activation layer for hierarchical probability modeling. While we have illustrated its application in medical imaging with a nnUNet model, the activation layer could be adapted to various hierarchical contexts and could be used with any model. The power of this framework lies in its ability to train a single segmentation model to recognize over 100 labels hierarchically. Throughout the training process, the loss function is computed over the whole tree, allowing for the optimization of each node at every stage of training. This hierarchical training ensures that the model respects the natural structure of the data, for instance, ensuring that the colon is identified within the abdominal cavity. Additionally, the proposed model takes an average of 35 ms per slice, meaning that a full whole-body CT scan with 1000 slices can be computed in 35 s, which is faster than existing models^2^.

In general, SALT consistently achieved relevant results for key organs: kidneys (Dice 0.92, except CT-ORG), liver (Dice > 0.95), spleen (Dice > 0.92), stomach (Dice > 0.9), and lungs (Dice > 0.91, except right middle lobe). The adrenal glands were the most challenging, with Dice values of 0.65–0.70, but performed well in terms of NSD (0.88–0.95). SALT’s segmentation included more contours, which reduced the Dice scores for smaller classes such as the adrenal glands. In the SAROS dataset, SALT achieved results comparable to an nnUNet-based network for BCA, with a notable improvement in the mediastinum class (0.95 vs. 0.84) and only minor performance differences (up to 0.05) in most classes, except for the brain (nnUNet 0.97 vs. SALT 0.75). Comparisons with TotalSegmentator showed similar performance, with Dice differences within 0.05 for most classes, except for adrenals and vena cava in FLARE22 and kidneys and bladder in CT-ORG. Larger errors occurred in complex gastrointestinal classes such as the intestine and duodenum, which are challenging due to their anatomical variability and unclear boundaries. Differences in Dice scores between datasets also reflect different annotation guidelines and the inherent variability of human anatomy, as shown in Fig. 7 and Table S8. TotalSegmentator results highlight these discrepancies, such as Dice scores for adrenal glands (0.835 in FLARE22 and 0.624 in WORD), illustrating dataset-specific challenges.

While several methods have explored hierarchical modeling in medical image segmentation, such as BayeSeg and other probabilistic graphical models, SALT offers a lightweight and easily integrable alternative. BayeSeg, for example, relies on structured probabilistic inference to model inter-class dependencies, which often requires additional architectural components or inference layers. In contrast, SALT enforces hierarchical consistency directly through a chained conditional softmax formulation, without modifying the network architecture or introducing inference-time complexity. This makes SALT particularly suitable for large-scale or high-resolution segmentation tasks, where computational simplicity and modular integration are critical. Therefore, SALT addresses a key limitation of traditional hierarchical models: the tendency to overpredict deep leaf nodes while still receiving partial credit due to shared ancestors. By enforcing conditional probability chaining along the hierarchy, SALT requires the model to be confident in all parent classes before assigning probability mass to a specific leaf node, thereby promoting hierarchical consistency and reducing incorrect “deep” predictions. However, this approach introduces a potential drawback. For nodes located deep in the hierarchy, the product of multiple conditional probabilities can lead to minimal final values, an effect analogous to vanishing probabilities, which may reduce the sharpness or certainty of predictions for fine-grained classes. This trade-off highlights the inherent tension between enforcing a strict hierarchical structure and preserving strong per-class confidence. It may need to be considered when applying SALT to tasks with particularly deep or imbalanced label trees.

The extraction of biomarkers from medical segmentations is an active field of research that has a variety of clinical applications, such as making accurate diagnoses^28^, monitoring a patient’s well-being^4,6^, or predicting overall survival^29,30^. This also includes BCA biomarkers, which are also relevant for disease progression^6^ and predicting patient survival outcomes^7,8^. Given the numerous applications and high volume of CT scans generated daily, models for segmentation and biomarker extraction must prioritize speed and efficiency. SALT’s fast processing suggests that, compared to other models, it could enable quicker biomarker extraction, resulting in increased throughput. This speed could facilitate its integration into clinical workflows, allowing the automatic calculation of essential biomarkers each time a patient undergoes a CT scan.

Although both TotalSegmentator (versions 1 and 2) and DynUNet + SALT rely on nnU-Net-based backbones, notable differences in inference speed were observed. These differences are primarily attributed to the self-configuring nature of TotalSegmentator, which involves additional runtime overhead from dynamic preprocessing, automatic architecture configuration, and multi-model ensemble handling. In contrast, DynUNet + SALT employs a fixed, lightweight architecture optimized explicitly for multi-organ segmentation, resulting in leaner and more predictable inference. Moreover, the SALT framework integrates hierarchical label prediction into a single conditional probability structure, reducing redundant computations and output channels. As shown in Fig. 4, Supplementary Table S7, and Supplementary Figure S2, SALT achieves consistently faster inference than both TotalSegmentator v1 and v2 across scan sizes, with the most considerable speed advantage observed for high-resolution CT volumes.

Although the per-scan speed improvement offered by SALT may appear modest (typically 1–2 min), it becomes highly relevant in real-world scenarios where segmentation is applied at scale. In radiology departments processing hundreds or thousands of scans per month, such time savings translate to significant reductions in GPU usage, energy consumption, and turnaround times. This efficiency is critical when segmentation models are deployed as background services to extract biomarkers for monitoring or population-scale analysis continuously. Even incremental gains in runtime efficiency contribute to system scalability and integration feasibility in both clinical and research environments. While the reported increases in accuracy and efficiency may appear modest in isolation, they carry meaningful implications for real-world deployment. In large-scale clinical environments, even minor improvements in runtime translate into substantial reductions in GPU load, energy consumption, and processing time across thousands of scans. More importantly, SALT introduces hierarchical consistency into segmentation outputs, ensuring anatomically plausible predictions that are especially valuable for downstream applications such as body composition analysis, organ-specific biomarker extraction, and vertebral-level reporting. These tasks require not only high segmentation accuracy but also structural validity to maintain clinical trust. From a translational perspective, methods like SALT, which improve consistency and efficiency without requiring changes to the network architecture, help bridge the gap between research models and scalable clinical integration.

A limitation of this study is SALT’s tendency to include organ and vessel walls in segmentations, differing from other datasets. While this can be advantageous for structures like bones (including cortical areas), it can introduce inaccuracies for others, like adrenal glands, by capturing abdominal fat. This may stem from annotation differences between SALT’s dataset (which includes walls) and TotalSegmentator. SALT also performed lower on aorta and vena cava segmentations (0.89 and 0.859 vs. 0.936 and 0.912 in TotalSegmentator) due to structural division requirements that may have confused. This limitation relates to SALT’s hierarchical tree model, which restricts each class to a single parent node. A graph-like structure with multiple parent nodes would improve anatomical representation but would alter softmax properties. Incorporating anatomical ontologies like the Foundational Model of Anatomy or SNOMED could standardize this hierarchy. Future work could explore multi-label segmentation to assign multiple classes to voxels, which is vital for overlapping structures like muscle and subcutaneous tissue. SALT’s compatibility with various datasets suggests potential for training with diverse sources without the need for dataset merging. Further refinement in segmentation to selectively include organ walls and the use of advanced post-processing could improve accuracy, supporting clinically valuable outcomes and addressing annotation consistency across datasets.

## Conclusion

In conclusion, the SALT framework leverages anatomical hierarchies to deliver efficient, comprehensive segmentations across 113 body regions, processing a full-body CT in 35 s, facilitating clinical integration and rapid biomarker computation. While primarily developed for CT body region segmentation, SALT’s activation function is adaptable to any hierarchical domain. Future work will aim to improve segmentation accuracy, explore graph-based structures for complex anatomies, and expand applications to multi-label segmentation and multi-dataset integration.

## Supplementary Information

Supplementary Information.

## Data Availability

The datasets used within this study are all publicly available and already published. In addition, the code for using SALT is publicly available under) (https://github.com/UMEssen/SALT).

## Abbreviations

BCA: Body composition analysis
CNN: Convolutional neural network
CT: Computed tomography
CT-ORG: CT volumes with multiple organ segmentations
(FLARE22): Fast and low-resource semi-supervised abdominal organ segmentation
FN: False negatives
FP: False positives
LCTSC: Lung CT segmentation challenge
LUNA16: Lung nodule analysis 2016
NSD: Normalized surface dice
SALT: Softmax for arbitrary label trees
SAROS: Sparsely annotated region and organ segmentation
TCIA: The cancer imaging archive
TP: True positives
WORD: Whole abdominal organ dataset
