# Supplementary material for: SALT: Introducing a framework for hierarchical segmentations in medical imaging using label trees
Source: Sci Rep. 2025 Dec 19;15:44140. doi: 10.1038/s41598-025-31639-1 (PMC12717041; doi:10.1038/s41598-025-31639-1)
Supplement: Supplementary file 1 — Supplementary Information. [file 41598_2025_31639_MOESM1_ESM.docx]

# Supplementary Materials


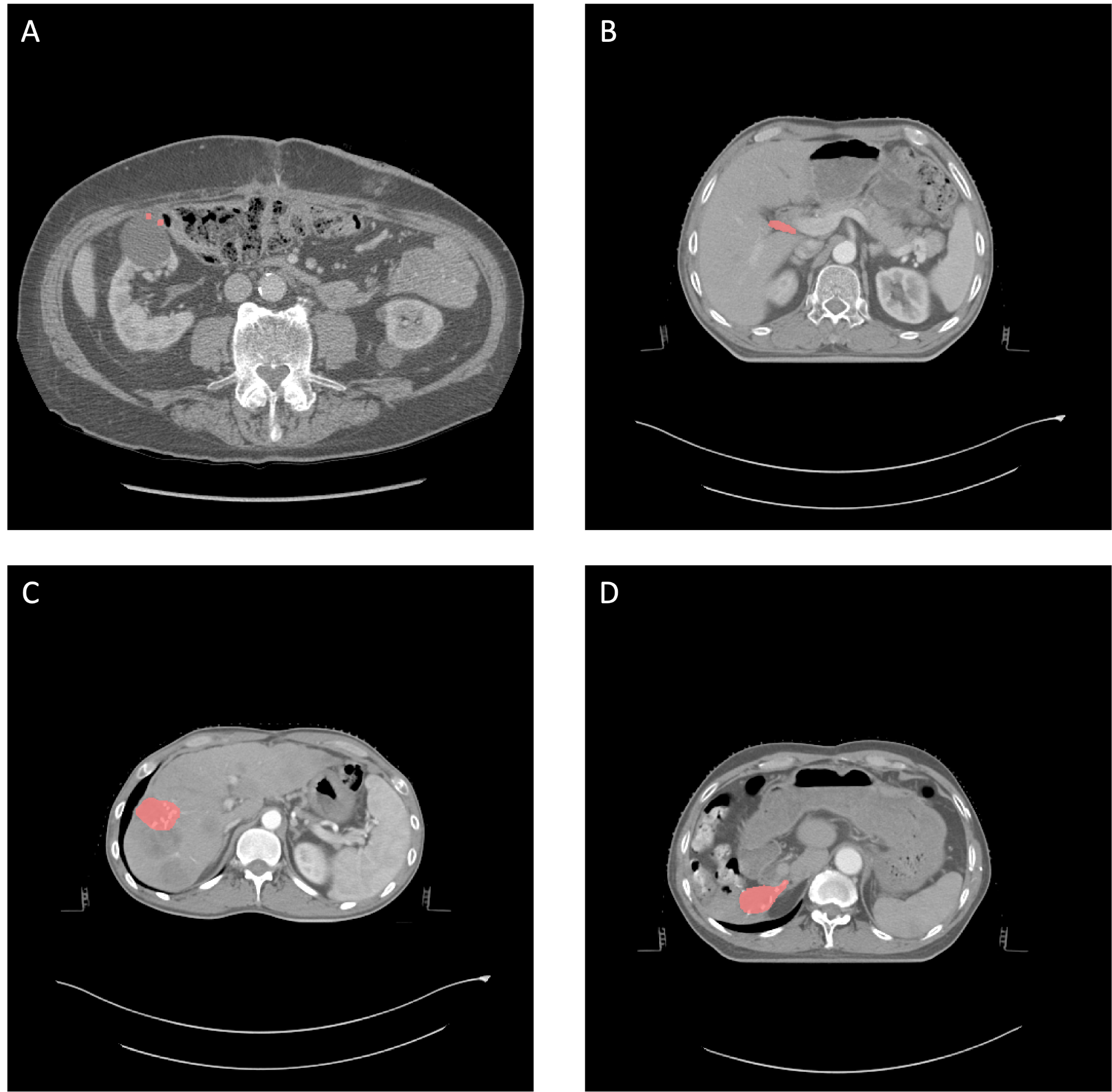


Figure S1: **Gallbladder Segmentations of the WORD dataset.** In A, five voxels were marked as gallbladder, and binary dilation was performed to visualize them. In B and D, the common bile duct was segmented instead of the gallbladder. In C, a tumor was identified as the gallbladder.


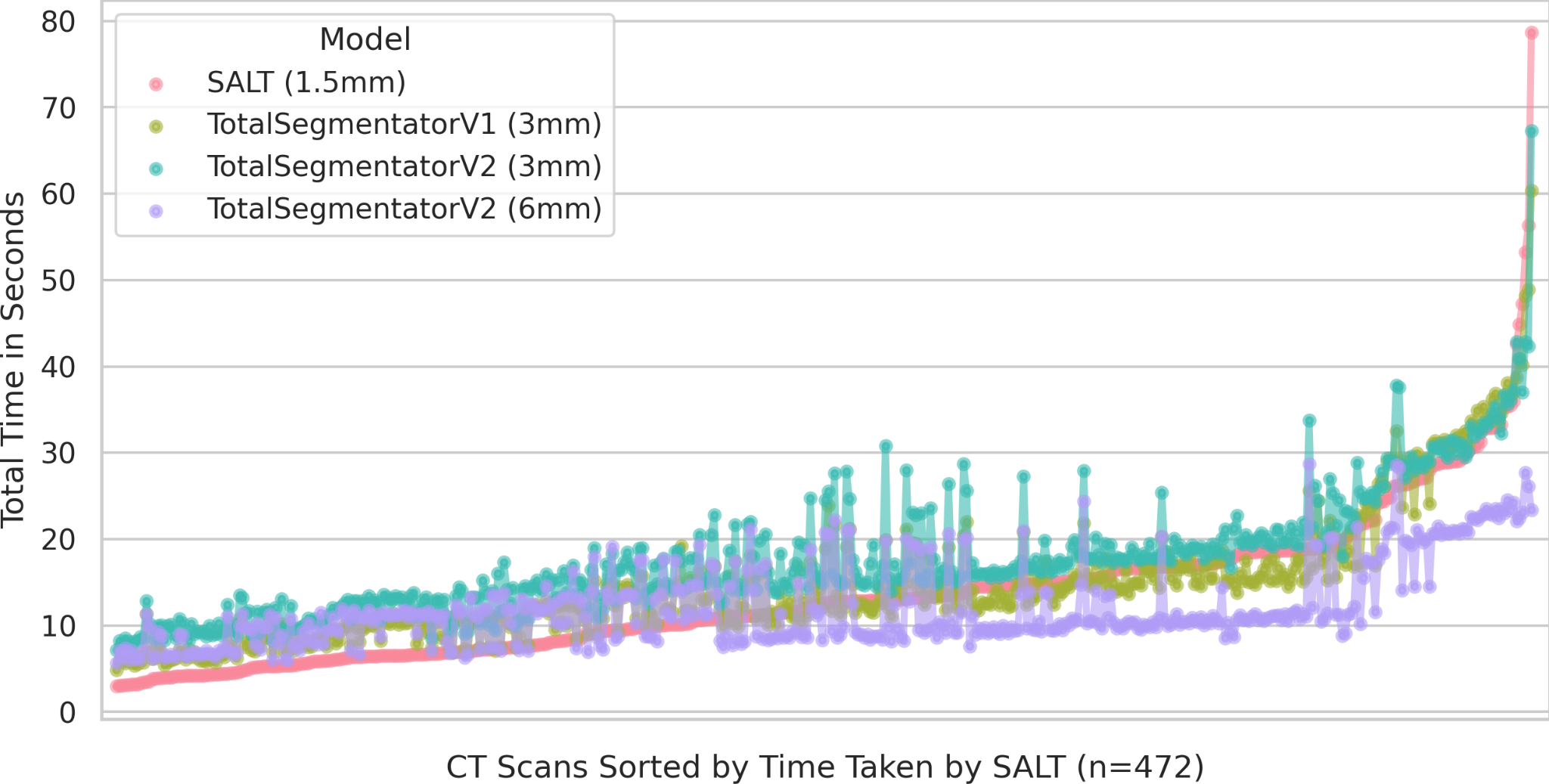


Figure S2: **Comparison between the speed of SALT and the TotalSegmentator**. The fast alternatives of the TotalSegmentator were run on the same set of 472 CT scans from Table 1. Version 1 offers a quick alternative using a single model with 3mm isotropic spacing, whereas Version 2 provides two options with isotropic spacing of 3mm and 6mm.

| **Label** | **SALT (Dice)** | **TSV2 (Dice)** | **SALT (NSD)** | **TSV2 (NSD)** |
| --- | --- | --- | --- | --- |
| Kidneys | 0.872  [0.854, 0.884] | 0.927  [0.912, 0.94] | 0.914  [0.884, 0.937] | 0.951  [0.92, 0.975] |
| Liver | 0.95  [0.946, 0.953] | 0.964  [0.961, 0.967] | 0.92  [0.906, 0.935] | 0.952  [0.945, 0.961] |
| Urinary Bladder | 0.829  [0.787, 0.867] | 0.881  [0.853, 0.905] | 0.731  [0.655, 0.805] | 0.858  [0.8, 0.906] |
| Bones | 0.872  [0.863, 0.879] | 0.877  [0.861, 0.89] | 0.919  [0.912, 0.926] | 0.883  [0.866, 0.897] |
| Brain | 0.486  [0, 0.973] | 0.488  [0, 0.976] | 0.49  [0, 0.981] | 0.492  [0, 0.985] |
| Lungs | 0.962  [0.958, 0.966] | 0.97  [0.964, 0.975] | 0.968  [0.951, 0.982] | 0.971  [0.955, 0.984] |

Table S1: **Dice scores and Normalized Surface Dice (NSD) for the CT-ORG dataset**. The scores are reported for SALT and Version 2 of the TotalSegmentator (TSV2). The 95% confidence intervals are reported in brackets.

| **Label** | **SALT (Dice)** | **TSV2 (Dice)** | **SALT (NSD)** | **TSV2 (NSD)** |
| --- | --- | --- | --- | --- |
| Adrenal Glands | 0.699  [0.683, 0.713] | 0.835  [0.825, 0.846] | 0.949  [0.935, 0.961] | 0.978  [0.969, 0.986] |
| Adrenal Gland (L) | 0.693  [0.674, 0.709] | 0.837  [0.824, 0.849] | 0.944  [0.925, 0.959] | 0.978  [0.965, 0.988] |
| Adrenal Gland (R) | 0.706  [0.687, 0.724] | 0.831  [0.814, 0.845] | 0.957  [0.944, 0.97] | 0.978  [0.965, 0.988] |
| Duodenum | 0.756  [0.74, 0.77] | 0.768  [0.749, 0.785] | 0.836  [0.821, 0.852] | 0.844  [0.828, 0.86] |
| Gallbladder | 0.861  [0.844, 0.877] | 0.894  [0.878, 0.91] | 0.956  [0.942, 0.969] | 0.958  [0.941, 0.973] |
| Kidneys | 0.92  [0.913, 0.925] | 0.915  [0.899, 0.929] | 0.959  [0.948, 0.968] | 0.938  [0.922, 0.953] |
| Kidney (L) | 0.919  [0.914, 0.924] | 0.914  [0.897, 0.928] | 0.963  [0.954, 0.97] | 0.94  [0.924, 0.955] |
| Kidney (R) | 0.921  [0.912, 0.928] | 0.921  [0.899, 0.936] | 0.956  [0.943, 0.967] | 0.939  [0.919, 0.955] |
| Liver | 0.952  [0.95, 0.954] | 0.972  [0.971, 0.973] | 0.966  [0.96, 0.97] | 0.977  [0.975, 0.979] |
| Pancreas | 0.843  [0.83, 0.856] | 0.833  [0.815, 0.85] | 0.934  [0.92, 0.946] | 0.929  [0.91, 0.945] |
| Spleen | 0.94  [0.935, 0.943] | 0.974  [0.972, 0.975] | 0.972  [0.966, 0.978] | 0.999  [0.998, 0.999] |
| Stomach | 0.93  [0.925, 0.935] | 0.954  [0.949, 0.958] | 0.974  [0.967, 0.979] | 0.982  [0.976, 0.987] |
| Aorta | 0.89  [0.882, 0.896] | 0.936  [0.926, 0.945] | 0.951  [0.937, 0.962] | 0.958  [0.94, 0.973] |
| Vena Cava Inferior | 0.859  [0.854, 0.865] | 0.912  [0.905, 0.918] | 0.942  [0.934, 0.949] | 0.954  [0.946, 0.96] |

Table S2: **Dice scores and Normalized Surface Dice (NSD) for the FLARE22 dataset**. The scores are reported for SALT and Version 2 of the TotalSegmentator (TSV2). The 95% confidence intervals are reported in brackets. (L) = Left, (R) = Right.

| **Label** | **SALT (Dice)** | **TSV2 (Dice)** | **SALT (NSD)** | **TSV2 (NSD)** |
| --- | --- | --- | --- | --- |
| Spinal Cord | 0.84  [0.833, 0.845] | 0.881  [0.874, 0.886] | 0.969  [0.963, 0.975] | 0.977  [0.971, 0.981] |
| Lungs | 0.941  [0.934, 0.947] | 0.959  [0.953, 0.965] | 0.894  [0.877, 0.909] | 0.937  [0.925, 0.949] |
| Lung (L) | 0.922  [0.9, 0.94] | 0.948  [0.933, 0.96] | 0.878  [0.849, 0.904] | 0.929  [0.908, 0.948] |
| Lung (R) | 0.946  [0.937, 0.952] | 0.961  [0.953, 0.967] | 0.896  [0.877, 0.913] | 0.938  [0.921, 0.952] |
| Pericardium | 0.894  [0.887, 0.901] | / | 0.792  [0.769, 0.815] | / |

Table S3: **Dice scores and Normalized Surface Dice (NSD) for the LCTSC dataset**. The scores are reported for SALT and Version 2 of the TotalSegmentator (TSV2). The 95% confidence intervals are reported in brackets. (L) = Left, (R) = Right.

| **Label** | **SALT (Dice)** | **TSV2 (Dice)** | **SALT (NSD)** | **TSV2 (NSD)** |
| --- | --- | --- | --- | --- |
| Lungs | 0.961  [0.956, 0.964] | 0.983  [0.981, 0.984] | 0.951  [0.935, 0.962] | 0.979  [0.976, 0.982] |
| Lung (L) | 0.956  [0.949, 0.961] | 0.982  [0.979, 0.984] | 0.945  [0.926, 0.958] | 0.981  [0.977, 0.983] |
| Lung Lower Lobe (L) | 0.931  [0.917, 0.94] | 0.961  [0.956, 0.966] | 0.905  [0.875, 0.927] | 0.953  [0.941, 0.964] |
| Lung Upper Lobe (L) | 0.948  [0.943, 0.952] | 0.969  [0.966, 0.971] | 0.943  [0.93, 0.953] | 0.962  [0.952, 0.97] |
| Lung (R) | 0.964  [0.96, 0.967] | 0.984  [0.982, 0.985] | 0.956  [0.943, 0.965] | 0.978  [0.975, 0.981] |
| Lung Lower Lobe (R) | 0.93  [0.918, 0.938] | 0.955  [0.948, 0.962] | 0.894  [0.871, 0.912] | 0.926  [0.913, 0.939] |
| Lung Middle Lobe (R) | 0.835  [0.787, 0.873] | 0.848  [0.8, 0.885] | 0.794  [0.75, 0.835] | 0.806  [0.763, 0.842] |
| Lung Upper Lobe (R) | 0.916  [0.894, 0.931] | 0.933  [0.911, 0.947] | 0.874  [0.845, 0.898] | 0.888  [0.862, 0.911] |

Table S4: **Dice scores and Normalized Surface Dice (NSD) for the LUNA16 dataset**. The scores are reported for SALT and Version 2 of the TotalSegmentator (TSV2). The 95% confidence intervals are reported in brackets. (L) = Left, (R) = Right.

| **Label** | **SALT (Dice)** | **TSV2 (Dice)** | **SALT (NSD)** | **TSV2 (NSD)** |
| --- | --- | --- | --- | --- |
| Abdominal Cavity | 0.98  [0.979, 0.981] | / | 0.995  [0.994, 0.995] | / |
| Bones | 0.911  [0.908, 0.914] | 0.805  [0.8, 0.811] | 0.991  [0.99, 0.992] | 0.942  [0.938, 0.945] |
| Brain | 0.758  [0.656, 0.858] | 0.939  [0.886, 0.974] | 0.794  [0.691, 0.896] | 0.964  [0.91, 0.996] |
| Muscles | 0.931  [0.928, 0.934] | / | 0.989  [0.987, 0.991] | / |
| Spinal Cord | 0.849  [0.845, 0.852] | 0.878  [0.872, 0.885] | 0.975  [0.972, 0.978] | 0.964  [0.958, 0.97] |
| Subcutaneous Tissue | 0.937  [0.932, 0.942] | / | 0.991  [0.989, 0.993] | / |
| Thoracic Cavity | 0.972  [0.969, 0.975] | / | 0.992  [0.99, 0.994] | / |
| Mediastinum | 0.95  [0.944, 0.955] | / | 0.981  [0.976, 0.984] | / |
| Pericardium | 0.952  [0.949, 0.955] | / | 0.982  [0.979, 0.984] | / |

Table S5: **Dice scores and Normalized Surface Dice (NSD) for the SAROS dataset**. The scores are reported for SALT and Version 2 of the TotalSegmentator (TSV2). The 95% confidence intervals are reported in brackets.

| **Label** | **SALT (Dice)** | **TSV2 (Dice)** | **SALT (NSD)** | **TSV2 (NSD)** |
| --- | --- | --- | --- | --- |
| Adrenal Glands | 0.65  [0.631, 0.667] | 0.624  [0.603, 0.643] | 0.887  [0.868, 0.903] | 0.856  [0.833, 0.875] |
| Colon | 0.808  [0.796, 0.819] | 0.773  [0.76, 0.785] | 0.828  [0.814, 0.841] | 0.784  [0.768, 0.798] |
| Duodenum | 0.642  [0.619, 0.662] | 0.623  [0.598, 0.644] | 0.741  [0.719, 0.761] | 0.718  [0.692, 0.74] |
| Kidneys | 0.923  [0.917, 0.926] | 0.925  [0.921, 0.928] | 0.97  [0.963, 0.974] | 0.973  [0.968, 0.977] |
| Kidney (L) | 0.922  [0.914, 0.928] | 0.922  [0.918, 0.926] | 0.971  [0.961, 0.978] | 0.969  [0.963, 0.974] |
| Kidney (R) | 0.922  [0.92, 0.925] | 0.927  [0.923, 0.931] | 0.969  [0.965, 0.972] | 0.976  [0.971, 0.98] |
| Liver | 0.951  [0.949, 0.953] | 0.956  [0.954, 0.957] | 0.952  [0.946, 0.958] | 0.96  [0.956, 0.963] |
| Pancreas | 0.802  [0.79, 0.812] | 0.791  [0.782, 0.8] | 0.922  [0.914, 0.93] | 0.911  [0.903, 0.918] |
| Small Bowel | 0.821  [0.81, 0.83] | 0.797  [0.784, 0.81] | 0.867  [0.855, 0.878] | 0.847  [0.831, 0.863] |
| Spleen | 0.927  [0.924, 0.93] | 0.939  [0.937, 0.942] | 0.962  [0.956, 0.968] | 0.986  [0.983, 0.989] |
| Stomach | 0.9  [0.89, 0.907] | 0.904  [0.898, 0.91] | 0.925  [0.914, 0.935] | 0.93  [0.922, 0.938] |
| Urinary Bladder | 0.864  [0.843, 0.882] | 0.903  [0.884, 0.918] | 0.914  [0.894, 0.931] | 0.96  [0.945, 0.971] |

Table S6: **Dice scores and Normalized Surface Dice (NSD) for the WORD dataset**. The scores are reported for SALT and Version 2 of the TotalSegmentator (TSV2). The 95% confidence intervals are reported in brackets. (L) = Left, (R) = Right.

|  | **CT-ORG** | **FLARE22** | **LCTSC** | **LUNA16** | **SAROS** | **WORD** |
| --- | --- | --- | --- | --- | --- | --- |
| **Inference Time** | 8.87 ± 11.88 | 2.84 ± 1.19 | 8.89 ± 2.84 | 2.74 ± 1.03 | 8.86 ± 7.68 | 10.25 ± 2.83 |
| **Total Time** | 17.73 ± 14.77 | 4.95 ± 1.46 | 13.21 ± 3.56 | 6.96 ± 2.01 | 15.95 ± 11.63 | 15.93 ± 3.03 |
| **Number of Slices** | 396.19 ± 187.16 | 167.64 ± 39.19 | 273.76 ± 37.0 | 208.68 ± 26.94 | 359.05 ± 183.77 | 385.44 ± 61.75 |
| **Seconds per Slice** | 0.04 ± 0.01 | 0.02 ± 0.008 | 0.04 ± 0.01 | 0.03 ± 0.009 | 0.04 ± 0.01 | 0.04 ± 0.003 |

Table S7. **Evaluation of the time taken by the model at inference time.** The inference and total time are reported in seconds, and all values are given as mean ± standard deviation. The times and the number of slices were stored after preprocessing the CT scan to a spacing of (1.5, 1.5, 1.5).

|  | Dice | NSD |
| --- | --- | --- |
| Adrenal Glands | FLARE22 vs. WORD: 0.0433* | FLARE22 vs. WORD: 2.6896e-06* |
| Bones | CT-ORG vs. SAROS: 2.1364e-09* | CT-ORG vs. SAROS: 4.2888e-12* |
| Brain | CT-ORG vs. SAROS: 1.0 | CT-ORG vs. SAROS: 1.0 |
| Duodenum | FLARE22 vs. WORD: 1.3189e-09* | FLARE22 vs. WORD: 1.5321e-06* |
| Kidney Left | FLARE22 vs. WORD: 0.2095 | FLARE22 vs. WORD: 0.0042* |
| Kidney Right | FLARE22 vs. WORD: 1.0 | FLARE22 vs. WORD: 0.8368 |
| Kidneys | CT-ORG vs. FLARE22: 1.7019e-06*  CT-ORG vs. WORD: 3.6064e-10*  FLARE22 vs. WORD: 1.0 | CT-ORG vs. FLARE22: 0.0050*  CT-ORG vs. WORD: 2.5901e-09*  FLARE22 vs. WORD: 0.1195 |
| Liver | CT-ORG vs. FLARE22: 1.0  CT-ORG vs. WORD: 1.0  FLARE22 vs. WORD: 1.0 | CT-ORG vs. FLARE22: 2.4277e-05*  CT-ORG vs. WORD: 0.0029*  FLARE22 vs. WORD: 1.0 |
| Lung Left | LCTSC vs. LUNA16: 1.0141e-05* | LCTSC vs. LUNA16: 6.1510e-06* |
| Lung Right | LCTSC vs. LUNA16: 1.9292e-05* | LCTSC vs. LUNA16: 1.3627e-08* |
| Lungs | CT-ORG vs. LCTSC: 0.0020*  CT-ORG vs. LUNA16: 1.0  LCTSC vs. LUNA16: 2.2457e-06* | CT-ORG vs. LCTSC: 3.8623e-08*  CT-ORG vs. LUNA16: 1.0  LCTSC vs. LUNA16: 1.1547e-06* |
| Pancreas | FLARE22 vs. WORD: 0.0006* | FLARE22 vs. WORD: 1.0 |
| Pericardium | LCTSC vs. SAROS: 1.1399e-23* | LCTSC vs. SAROS: 1.5610e-27* |
| Spinal Cord | LCTSC vs. SAROS: 0.5081 | LCTSC vs. SAROS: 1.0 |
| Spleen | FLARE22 vs. WORD: 0.0001* | FLARE22 vs. WORD: 1.0 |
| Stomach | FLARE22 vs. WORD: 3.3810e-06* | FLARE22 vs. WORD: 2.4462e-09* |
| Urinary Bladder | CT-ORG vs. WORD: 0.1782 | CT-ORG vs. WORD: 5.6032e-06* |

Table S8. **Results of the statistical tests for the Dice score and the Normalized Surface Dice (NSD) across the datasets**. For organs present in multiple datasets, the results across these datasets were compared using statistical tests. The comparisons were conducted either using the Mann-Whitney U test (for labels belonging to two datasets) or with the Kruskal-Wallis test with Dunn’s post-hoc multiple comparison test adjustment (for more than two datasets). Moreover, the p-values were adjusted for multiple comparisons using Bonferroni’s method. A star (*) denotes statistically significant differences in results between the reported datasets for a particular organ label. In all cases, p-values less than or equal to 0.05 were considered significant.

| Leaf Labels | Train | Val | Test |
| --- | --- | --- | --- |
| body,subcutaneous_tissue | 600 | 150 | 150 |
| body,muscles,other | 600 | 150 | 150 |
| body,abdominal_cavity,other | 600 | 150 | 150 |
| body,thoracic_cavity,other | 600 | 150 | 150 |
| body,bones,other | 600 | 150 | 150 |
| body,thoracic_cavity,mediastinum,pericardium,other | 594 | 143 | 147 |
| body,thoracic_cavity,mediastinum,other | 600 | 150 | 150 |
| body,spinal_cord | 600 | 150 | 150 |
| body,abdominal_cavity,spleen | 598 | 149 | 149 |
| body,abdominal_cavity,kidneys,kidney_right | 568 | 143 | 144 |
| body,abdominal_cavity,kidneys,kidney_left | 583 | 146 | 146 |
| body,abdominal_cavity,gallbladder | 486 | 122 | 127 |
| body,abdominal_cavity,liver | 600 | 150 | 150 |
| body,abdominal_cavity,stomach | 600 | 150 | 150 |
| body,thoracic_cavity,mediastinum,aorta_thoracica_pass_mediastinum | 600 | 150 | 150 |
| body,thoracic_cavity,mediastinum,vci_pass_thoracica | 595 | 149 | 150 |
| body,abdominal_cavity,portal_vein_and_splenic_vein | 589 | 149 | 149 |
| body,abdominal_cavity,pancreas | 589 | 147 | 148 |
| body,abdominal_cavity,adrenal_glands,adrenal_gland_right | 592 | 148 | 149 |
| body,abdominal_cavity,adrenal_glands,adrenal_gland_left | 590 | 147 | 148 |
| body,thoracic_cavity,lungs,lung_left,lung_upper_lobe_left | 593 | 147 | 149 |
| body,thoracic_cavity,lungs,lung_left,lung_lower_lobe_left | 599 | 150 | 150 |
| body,thoracic_cavity,lungs,lung_right,lung_upper_lobe_right | 487 | 119 | 127 |
| body,thoracic_cavity,lungs,lung_right,lung_middle_lobe_right | 593 | 144 | 147 |
| body,thoracic_cavity,lungs,lung_right,lung_lower_lobe_right | 599 | 150 | 150 |
| body,bones,spine,lumbar_spine,vertebrae_L5 | 372 | 92 | 94 |
| body,bones,spine,lumbar_spine,vertebrae_L4 | 406 | 104 | 101 |
| body,bones,spine,lumbar_spine,vertebrae_L3 | 449 | 117 | 116 |
| body,bones,spine,lumbar_spine,vertebrae_L2 | 523 | 135 | 134 |
| body,bones,spine,lumbar_spine,vertebrae_L1 | 575 | 145 | 143 |
| body,bones,spine,thoracic_spine,vertebrae_T12 | 594 | 147 | 148 |
| body,bones,spine,thoracic_spine,vertebrae_T11 | 600 | 150 | 149 |
| body,bones,spine,thoracic_spine,vertebrae_T10 | 594 | 146 | 147 |
| body,bones,spine,thoracic_spine,vertebrae_T9 | 578 | 142 | 145 |
| body,bones,spine,thoracic_spine,vertebrae_T8 | 540 | 135 | 141 |
| body,bones,spine,thoracic_spine,vertebrae_T7 | 490 | 117 | 133 |
| body,bones,spine,thoracic_spine,vertebrae_T6 | 434 | 104 | 115 |
| body,bones,spine,thoracic_spine,vertebrae_T5 | 407 | 100 | 102 |
| body,bones,spine,thoracic_spine,vertebrae_T4 | 402 | 99 | 100 |
| body,bones,spine,thoracic_spine,vertebrae_T3 | 401 | 99 | 100 |
| body,bones,spine,thoracic_spine,vertebrae_T2 | 401 | 99 | 100 |
| body,bones,spine,thoracic_spine,vertebrae_T1 | 400 | 99 | 100 |
| body,bones,spine,cervical_spine,vertebrae_C7 | 396 | 97 | 98 |
| body,bones,spine,cervical_spine,vertebrae_C6 | 338 | 82 | 83 |
| body,bones,spine,cervical_spine,vertebrae_C5 | 245 | 67 | 62 |
| body,bones,spine,cervical_spine,vertebrae_C4 | 206 | 53 | 53 |
| body,bones,spine,cervical_spine,vertebrae_C3 | 192 | 51 | 46 |
| body,bones,spine,cervical_spine,vertebrae_C2 | 187 | 50 | 44 |
| body,bones,spine,cervical_spine,vertebrae_C1 | 184 | 45 | 42 |
| body,thoracic_cavity,mediastinum,pericardium,heart_myocardium | 594 | 144 | 148 |
| body,thoracic_cavity,mediastinum,pericardium,heart_atrium_left | 563 | 140 | 145 |
| body,thoracic_cavity,mediastinum,pericardium,heart_ventricle_left | 591 | 143 | 147 |
| body,thoracic_cavity,mediastinum,pericardium,heart_atrium_right | 589 | 143 | 147 |
| body,thoracic_cavity,mediastinum,pericardium,heart_ventricle_right | 594 | 144 | 148 |
| body,thoracic_cavity,mediastinum,pulmonary_artery_pass_mediastinum | 420 | 102 | 111 |
| body,brain | 184 | 41 | 43 |
| body,abdominal_cavity,iliac_arteries,iliac_artery_left | 409 | 101 | 101 |
| body,abdominal_cavity,iliac_arteries,iliac_artery_right | 387 | 96 | 98 |
| body,abdominal_cavity,iliac_venae,iliac_vena_left | 369 | 90 | 93 |
| body,abdominal_cavity,iliac_venae,iliac_vena_right | 378 | 95 | 96 |
| body,abdominal_cavity,small_bowel | 559 | 138 | 140 |
| body,abdominal_cavity,duodenum | 553 | 137 | 141 |
| body,abdominal_cavity,colon | 589 | 142 | 149 |
| body,bones,rib_cage,rib_cage_left,rib_left_1 | 401 | 98 | 100 |
| body,bones,rib_cage,rib_cage_left,rib_left_2 | 401 | 98 | 100 |
| body,bones,rib_cage,rib_cage_left,rib_left_3 | 404 | 99 | 100 |
| body,bones,rib_cage,rib_cage_left,rib_left_4 | 425 | 108 | 110 |
| body,bones,rib_cage,rib_cage_left,rib_left_5 | 511 | 127 | 133 |
| body,bones,rib_cage,rib_cage_left,rib_left_6 | 587 | 142 | 145 |
| body,bones,rib_cage,rib_cage_left,rib_left_7 | 594 | 147 | 149 |
| body,bones,rib_cage,rib_cage_left,rib_left_8 | 597 | 149 | 149 |
| body,bones,rib_cage,rib_cage_left,rib_left_9 | 599 | 150 | 150 |
| body,bones,rib_cage,rib_cage_left,rib_left_10 | 600 | 150 | 150 |
| body,bones,rib_cage,rib_cage_left,rib_left_11 | 599 | 149 | 150 |
| body,bones,rib_cage,rib_cage_left,rib_left_12 | 572 | 143 | 146 |
| body,bones,rib_cage,rib_cage_right,rib_right_1 | 401 | 98 | 100 |
| body,bones,rib_cage,rib_cage_right,rib_right_2 | 401 | 98 | 100 |
| body,bones,rib_cage,rib_cage_right,rib_right_3 | 403 | 99 | 101 |
| body,bones,rib_cage,rib_cage_right,rib_right_4 | 428 | 108 | 109 |
| body,bones,rib_cage,rib_cage_right,rib_right_5 | 507 | 127 | 128 |
| body,bones,rib_cage,rib_cage_right,rib_right_6 | 585 | 144 | 145 |
| body,bones,rib_cage,rib_cage_right,rib_right_7 | 595 | 146 | 149 |
| body,bones,rib_cage,rib_cage_right,rib_right_8 | 597 | 149 | 149 |
| body,bones,rib_cage,rib_cage_right,rib_right_9 | 598 | 150 | 150 |
| body,bones,rib_cage,rib_cage_right,rib_right_10 | 600 | 150 | 150 |
| body,bones,rib_cage,rib_cage_right,rib_right_11 | 598 | 148 | 150 |
| body,bones,rib_cage,rib_cage_right,rib_right_12 | 572 | 144 | 143 |
| body,bones,humeri,humerus_left | 432 | 103 | 108 |
| body,bones,humeri,humerus_right | 446 | 107 | 115 |
| body,bones,scapulae,scapula_left | 466 | 111 | 124 |
| body,bones,scapulae,scapula_right | 460 | 113 | 124 |
| body,bones,claviculae,clavicula_left | 402 | 99 | 101 |
| body,bones,claviculae,clavicula_right | 402 | 99 | 100 |
| body,bones,femora,femur_left | 354 | 84 | 91 |
| body,bones,femora,femur_right | 293 | 75 | 78 |
| body,bones,hips,hip_left | 371 | 90 | 92 |
| body,bones,hips,hip_right | 368 | 91 | 92 |
| body,bones,sacrum | 348 | 84 | 89 |
| body,abdominal_cavity,urinary_bladder | 292 | 79 | 80 |
| body,muscles,gluteus_maximi,gluteus_maximus_left | 320 | 82 | 86 |
| body,muscles,gluteus_maximi,gluteus_maximus_right | 328 | 82 | 86 |
| body,muscles,gluteus_medii,gluteus_medius_left | 357 | 88 | 90 |
| body,muscles,gluteus_medii,gluteus_medius_right | 363 | 89 | 90 |
| body,muscles,gluteus_minimi,gluteus_minimus_left | 306 | 82 | 82 |
| body,muscles,gluteus_minimi,gluteus_minimus_right | 310 | 82 | 83 |
| body,muscles,autochthone,autochthon_left | 600 | 150 | 150 |
| body,muscles,autochthone,autochthon_right | 600 | 150 | 150 |
| body,muscles,iliopsoai,iliopsoas_left | 552 | 142 | 138 |
| body,muscles,iliopsoai,iliopsoas_right | 555 | 143 | 138 |
| body,abdominal_cavity,aorta_abdominalis | 600 | 149 | 150 |
| body,thoracic_cavity,mediastinum,pericardium,aorta_thoracica_pass_pericardium | 455 | 113 | 122 |
| body,abdominal_cavity,vci_pass_abdominalis | 600 | 150 | 150 |
| body,thoracic_cavity,mediastinum,pericardium,pulmonary_artery_pass_pericardium | 411 | 101 | 105 |

Table S9. **Leaf-level anatomical label occurrence across dataset splits.** Distribution of all leaf-level segmentation classes occurrences for the training, validation, and test sets of the SAROS dataset. Values indicate the number of CT scans in which each anatomical structure is present at least once, based on binary per-scan occurrence.

| Internal Hierarchical Labels | Train | Val | Test |
| --- | --- | --- | --- |
| body | 600 | 150 | 150 |
| body,muscles | 600 | 150 | 150 |
| body,abdominal_cavity | 600 | 150 | 150 |
| body,thoracic_cavity | 600 | 150 | 150 |
| body,bones | 600 | 150 | 150 |
| body,thoracic_cavity,mediastinum | 600 | 150 | 150 |
| body,thoracic_cavity,mediastinum,pericardium | 594 | 145 | 148 |
| body,abdominal_cavity,kidneys | 587 | 147 | 148 |
| body,abdominal_cavity,adrenal_glands | 596 | 148 | 149 |
| body,thoracic_cavity,lungs | 599 | 150 | 150 |
| body,thoracic_cavity,lungs,lung_left | 599 | 150 | 150 |
| body,thoracic_cavity,lungs,lung_right | 599 | 150 | 150 |
| body,bones,spine | 600 | 150 | 150 |
| body,bones,spine,lumbar_spine | 575 | 145 | 143 |
| body,bones,spine,thoracic_spine | 600 | 150 | 150 |
| body,bones,spine,cervical_spine | 397 | 97 | 98 |
| body,abdominal_cavity,iliac_arteries | 409 | 101 | 101 |
| body,abdominal_cavity,iliac_venae | 380 | 95 | 96 |
| body,bones,rib_cage | 600 | 150 | 150 |
| body,bones,rib_cage,rib_cage_left | 600 | 150 | 150 |
| body,bones,rib_cage,rib_cage_right | 600 | 150 | 150 |
| body,bones,humeri | 451 | 108 | 116 |
| body,bones,scapulae | 475 | 115 | 128 |
| body,bones,claviculae | 403 | 99 | 101 |
| body,bones,femora | 356 | 84 | 91 |
| body,bones,hips | 373 | 92 | 92 |
| body,muscles,gluteus_maximi | 328 | 82 | 86 |
| body,muscles,gluteus_medii | 366 | 89 | 90 |
| body,muscles,gluteus_minimi | 311 | 82 | 84 |
| body,muscles,autochthone | 600 | 150 | 150 |
| body,muscles,iliopsoai | 560 | 143 | 138 |

Table S10. **Coverage of hierarchical internal anatomical labels across dataset splits.** Overview of the internal class distribution to intermediate nodes within the hierarchical anatomical label structure of the SAROS dataset. The coverage was computed by aggregating all descendant leaf labels and counting the number of CT scans that contain at least one associated structure.

##

## Implementation Details (T1)

To evaluate the model's performance during training, a different Dice score formulation was devised. This approach uses the tree structure to create an encoding for each node, thereby eliminating the need to merge masks for evaluation purposes. The standard definition of Dice score can be computed using true positives (TP), false positives (FP), and false negatives (FN):

[
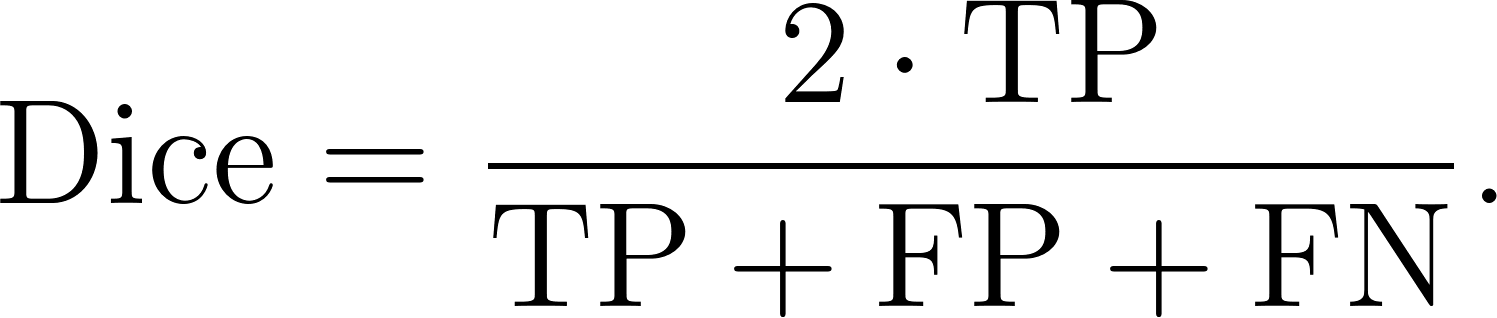
](https://www.codecogs.com/eqnedit.php?latex=%5Ctext%7BDice%7D%20%3D%20%5Cfrac%7B2%5Ccdot%20%5Ctext%7BTP%7D%7D%7B%5Ctext%7BTP%7D%2B%5Ctext%7BFP%7D%2B%5Ctext%7BFN%7D%7D.#0)

Let [
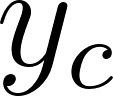
](https://www.codecogs.com/eqnedit.php?latex=y_c#0) and [
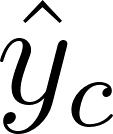
](https://www.codecogs.com/eqnedit.php?latex=%5Chat%7By%7D_c#0) be binary vectors representing the ground truth and the prediction for a specific class [
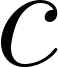
](https://www.codecogs.com/eqnedit.php?latex=c#0). Then TP, FP, and FN can be computed for the same class [
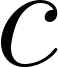
](https://www.codecogs.com/eqnedit.php?latex=c#0) using simple logical operations such as a logical and ([
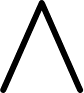
](https://www.codecogs.com/eqnedit.php?latex=%5Cland#0)) and logical not ([
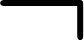
](https://www.codecogs.com/eqnedit.php?latex=%5Cneg#0)) and by computing the bit summation over each voxel [
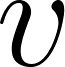
](https://www.codecogs.com/eqnedit.php?latex=v#0) in the voxels set [
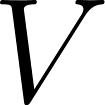
](https://www.codecogs.com/eqnedit.php?latex=V#0):

[
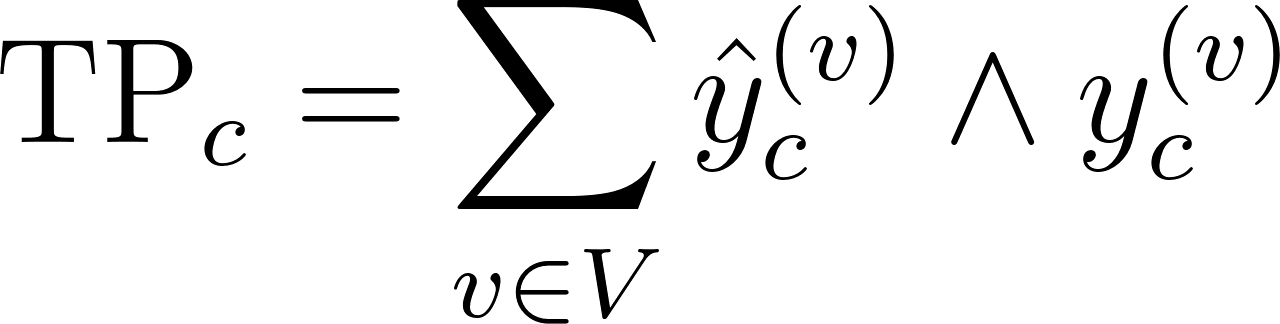
](https://www.codecogs.com/eqnedit.php?latex=%5Ctext%7BTP%7D_c%3D%5Csum_%7Bv%20%5Cin%20V%7D%5Chat%7By%7D%5E%7B(v)%7D_c%5Cland%20y%5E%7B(v)%7D_c#0)

[
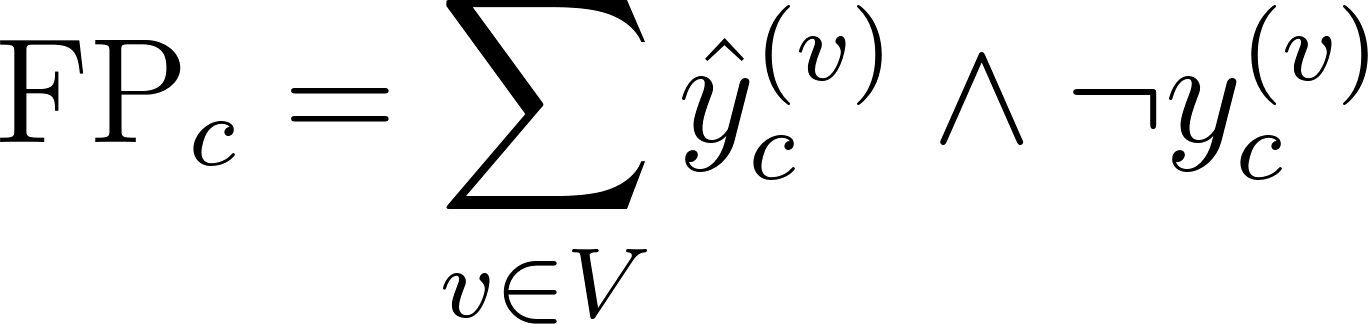
](https://www.codecogs.com/eqnedit.php?latex=%5Ctext%7BFP%7D_c%3D%5Csum_%7Bv%20%5Cin%20V%7D%5Chat%7By%7D%5E%7B(v)%7D_c%5Cland%20%5Cneg%20y%5E%7B(v)%7D_c#0)

[
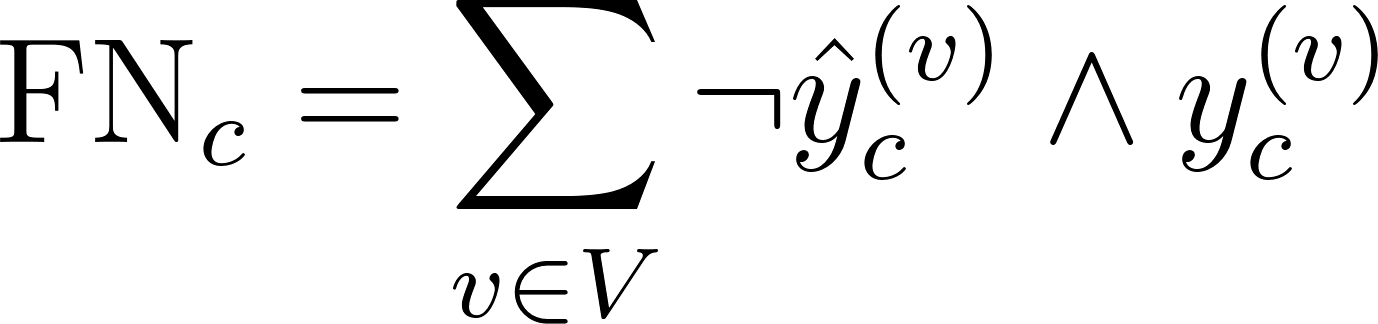
](https://www.codecogs.com/eqnedit.php?latex=%5Ctext%7BFN%7D_c%3D%5Csum_%7Bv%20%5Cin%20V%7D%5Cneg%5Chat%7By%7D%5E%7B(v)%7D_c%5Cland%20y%5E%7B(v)%7D_c#0)

To effectively assess the hierarchical relationships among classes, each class is assigned a bitwise encoding. The size of the encoding depends on the amount of bytes needed for uniquely encoding the node relationships. This means that the size of the encoding is [
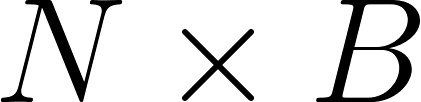
](https://www.codecogs.com/eqnedit.php?latex=N%20%5Ctimes%20B#0), where [
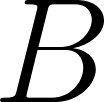
](https://www.codecogs.com/eqnedit.php?latex=B#0) is the number of bytes. The bitwise encoding at position [
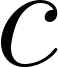
](https://www.codecogs.com/eqnedit.php?latex=c#0) of the matrix has size [
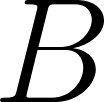
](https://www.codecogs.com/eqnedit.php?latex=B#0) and it is a signature that represents which nodes are traversed to get to node [
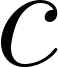
](https://www.codecogs.com/eqnedit.php?latex=c#0), i.e., all the parents of the node. Additionally, a bitwise mask of size [
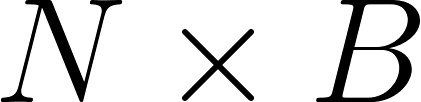
](https://www.codecogs.com/eqnedit.php?latex=N%20%5Ctimes%20B#0) is also computed to mask the relevant bits of the encoding that correspond to the parents and the siblings of each node [
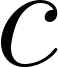
](https://www.codecogs.com/eqnedit.php?latex=c#0). For bitwise signatures with no more than 64 bits of encoding, native data types from the numpy package can be employed. In the other cases, the encoding can be split into individual bytes, and it requires an additional boolean logical and reduction operation to check for a binary match. Using these two matrices, it is possible to compute the Dice for voxel [
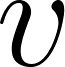
](https://www.codecogs.com/eqnedit.php?latex=v#0) and class [
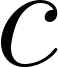
](https://www.codecogs.com/eqnedit.php?latex=c#0) by converting the ground truth ([
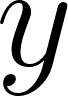
](https://www.codecogs.com/eqnedit.php?latex=y#0)) and the prediction ([
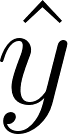
](https://www.codecogs.com/eqnedit.php?latex=%5Chat%7By%7D#0)) to a binary representation that uses these encodings. Let [
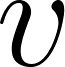
](https://www.codecogs.com/eqnedit.php?latex=v#0) be a voxel of the ground truth [
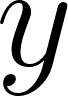
](https://www.codecogs.com/eqnedit.php?latex=y#0) or of the prediction [
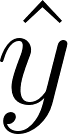
](https://www.codecogs.com/eqnedit.php?latex=%5Chat%7By%7D#0), and let [
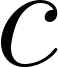
](https://www.codecogs.com/eqnedit.php?latex=c#0) be the class for which the Dice is computed. To compute the binary representation [
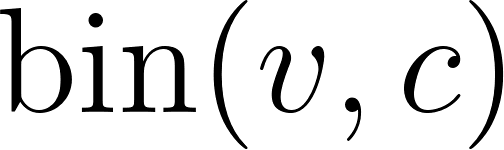
](https://www.codecogs.com/eqnedit.php?latex=%5Ctext%7Bbin%7D(v%2C%20c)#0), the voxel [
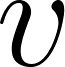
](https://www.codecogs.com/eqnedit.php?latex=v#0) is first converted to its bit encoding, and then it is masked using the bitwise mask of class [
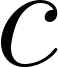
](https://www.codecogs.com/eqnedit.php?latex=c#0):

[
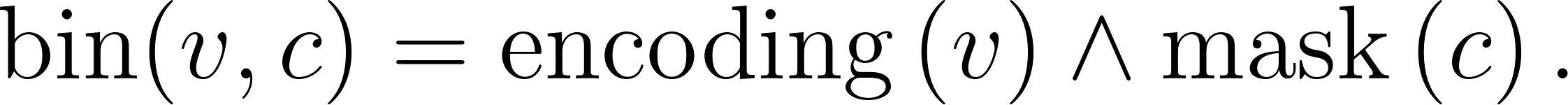
](https://www.codecogs.com/eqnedit.php?latex=%5Ctext%7Bbin%7D(v%2C%20c)%20%3D%20%5Ctext%7Bencoding%7D%5Cleft(v%5Cright)%5Cland%5Ctext%7Bmask%7D%5Cleft(c%5Cright).#0)

The result is a binary representation of the hierarchical affiliation of the voxel [
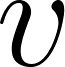
](https://www.codecogs.com/eqnedit.php?latex=v#0) to the class [
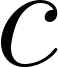
](https://www.codecogs.com/eqnedit.php?latex=c#0). In practice, this is done for a set of voxels [
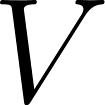
](https://www.codecogs.com/eqnedit.php?latex=V#0) at the same time, resulting in a two-dimensional array of binary representations of each voxel [
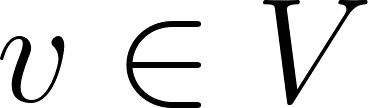
](https://www.codecogs.com/eqnedit.php?latex=v%20%5Cin%20V#0). The binary representation is then compared to the binary encoding [
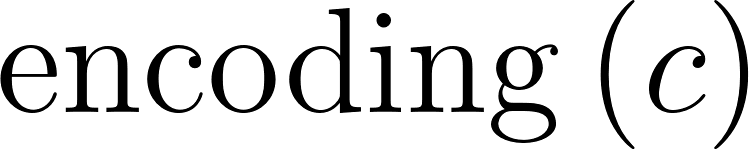
](https://www.codecogs.com/eqnedit.php?latex=%5Ctext%7Bencoding%7D%5Cleft(c%5Cright)#0) of class [
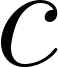
](https://www.codecogs.com/eqnedit.php?latex=c#0) with an equality operator ([
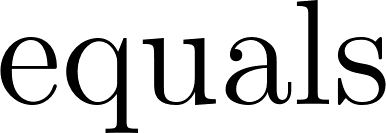
](https://www.codecogs.com/eqnedit.php?latex=%5Ctext%7Bequals%7D#0)):

[
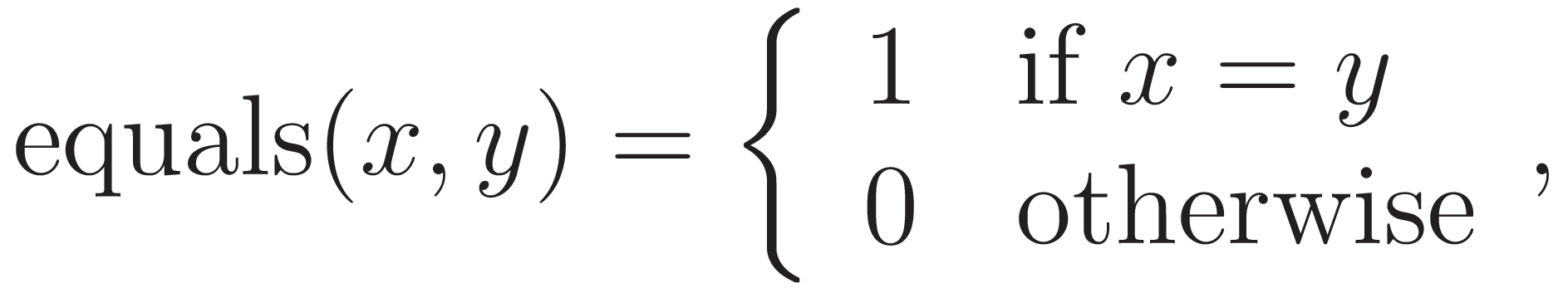
](http://www.sciweavers.org/tex2img.php?bc=Transparent&fc=Black&im=jpg&fs=100&ff=modern&edit=0&eq=%5Ctext%7Bequals%7D(x%2C%20y)%20%3D%20%5Cleft%5C%7B%5Cbegin%7Barray%7D%7Bll%7D1%20%20%26%20%5Cmbox%7Bif%20%7D%20x%20%3D%20y%20%5C%5C0%20%26%20%5Cmbox%7Botherwise%7D%5Cend%7Barray%7D%2C#0)

and the ground truth and the prediction are redefined as:

[**
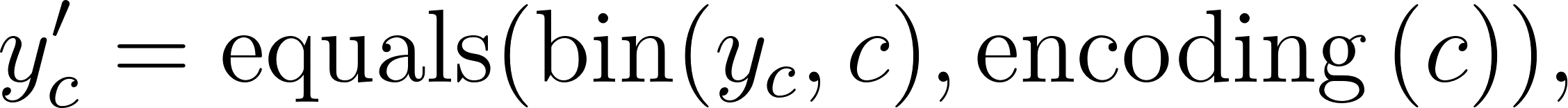
**](https://www.codecogs.com/eqnedit.php?latex=y'_c%20%3D%20%5Ctext%7Bequals%7D(%5Ctext%7Bbin%7D(y_c%2C%20c)%2C%20%5Ctext%7Bencoding%7D%5Cleft(c%5Cright))%2C#0)

[
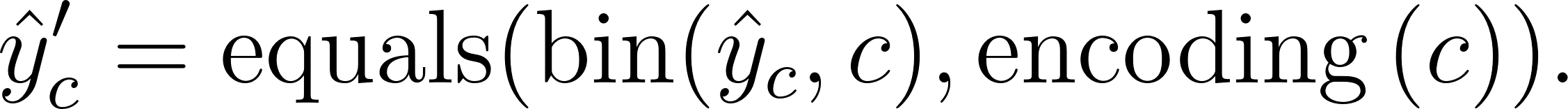
](https://www.codecogs.com/eqnedit.php?latex=%5Chat%7By%7D'_c%20%3D%20%5Ctext%7Bequals%7D(%5Ctext%7Bbin%7D(%5Chat%7By%7D_c%2C%20c)%2C%5Ctext%7Bencoding%7D%5Cleft(c%5Cright)).#0)

Now, [
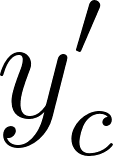
](https://www.codecogs.com/eqnedit.php?latex=y'_c#0) and [
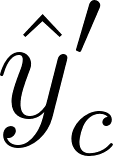
](https://www.codecogs.com/eqnedit.php?latex=%5Chat%7By%7D'_c#0) can be used to compute the Dice score with the true positive, false negative, and false positive formulas shown above.

## Reachability Matrix (T2)

Let [
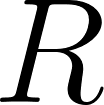
](https://www.codecogs.com/eqnedit.php?latex=R#0) be the reachability matrix of size [
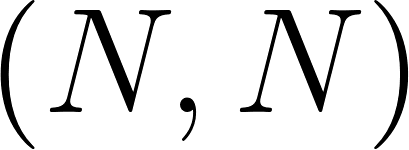
](https://www.codecogs.com/eqnedit.php?latex=(N%2CN)#0), where [
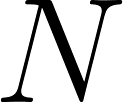
](https://www.codecogs.com/eqnedit.php?latex=N#0) is the number of nodes in the tree, and let [
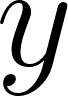
](https://www.codecogs.com/eqnedit.php?latex=y#0) be the ground truth label with [
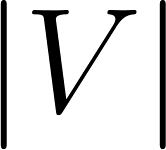
](https://www.codecogs.com/eqnedit.php?latex=%7CV%7C#0) elements (each corresponding to a voxel's label). We construct a new label [
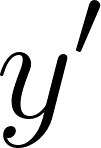
](https://www.codecogs.com/eqnedit.php?latex=y'#0) of size [
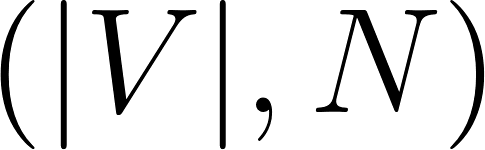
](https://www.codecogs.com/eqnedit.php?latex=(%7CV%7C%2C%20N)#0) by indexing the reachability matrix [
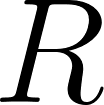
](https://www.codecogs.com/eqnedit.php?latex=R#0) using the ground truth [
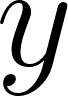
](https://www.codecogs.com/eqnedit.php?latex=y#0). This operation maps each voxel label to its corresponding encoding column in [
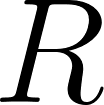
](https://www.codecogs.com/eqnedit.php?latex=R#0), or in other words, [
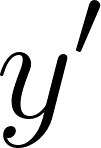
](https://www.codecogs.com/eqnedit.php?latex=y'#0) corresponds to the traversed nodes from the root to the voxel's label. Considering the prediction [
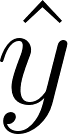
](https://www.codecogs.com/eqnedit.php?latex=%5Chat%7By%7D#0) of size [
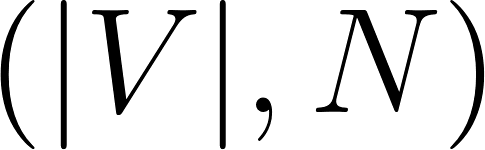
](https://www.codecogs.com/eqnedit.php?latex=(%7CV%7C%2C%20N)#0), the cross-entropy and the Dice loss can be computed using [
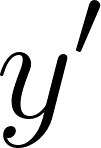
](https://www.codecogs.com/eqnedit.php?latex=y'#0) and [
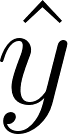
](https://www.codecogs.com/eqnedit.php?latex=%5Chat%7By%7D#0). Unlike the conventional softmax approach, this method optimizes each node along with all its ancestors, which is a result of the label encoding and the implementation of chained conditional probabilities within the activation layers.

## Modeling Conditional Probabilities for Trees (T3)

An arbitrary tree can be defined by its adjacency matrix [
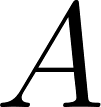
](https://www.codecogs.com/eqnedit.php?latex=A#0) of size [
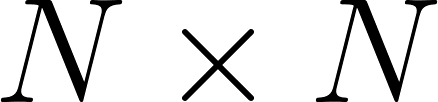
](https://www.codecogs.com/eqnedit.php?latex=N%20%5Ctimes%20N#0), where [
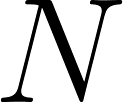
](https://www.codecogs.com/eqnedit.php?latex=N#0) is the number of nodes and each value [
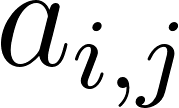
](https://www.codecogs.com/eqnedit.php?latex=a_%7Bi%2Cj%7D#0) of the matrix represents an edge between parent node [
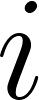
](https://www.codecogs.com/eqnedit.php?latex=i#0) and child node [
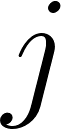
](https://www.codecogs.com/eqnedit.php?latex=j#0). Since the tree is unweighted, [
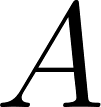
](https://www.codecogs.com/eqnedit.php?latex=A#0) has only binary elements, and [
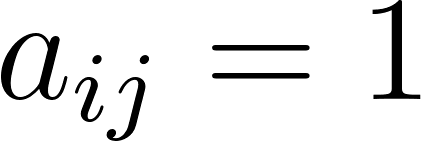
](https://www.codecogs.com/eqnedit.php?latex=a_%7Bij%7D%20%3D%201#0) represents a connection between parent node [
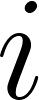
](https://www.codecogs.com/eqnedit.php?latex=i#0) and child node [
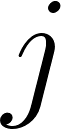
](https://www.codecogs.com/eqnedit.php?latex=j#0). Another useful structure is the reachability matrix [
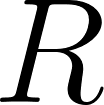
](https://www.codecogs.com/eqnedit.php?latex=R#0), which encodes in row [
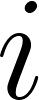
](https://www.codecogs.com/eqnedit.php?latex=i#0) all the nodes [
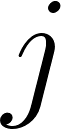
](https://www.codecogs.com/eqnedit.php?latex=j#0) that can be reached from node [
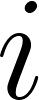
](https://www.codecogs.com/eqnedit.php?latex=i#0), and in column [
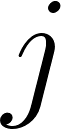
](https://www.codecogs.com/eqnedit.php?latex=j#0) all the traversed nodes between the root and node [
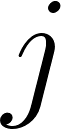
](https://www.codecogs.com/eqnedit.php?latex=j#0). This matrix is also binary and it can be derived from the adjacency matrix using matrix multiplications and additions:

[
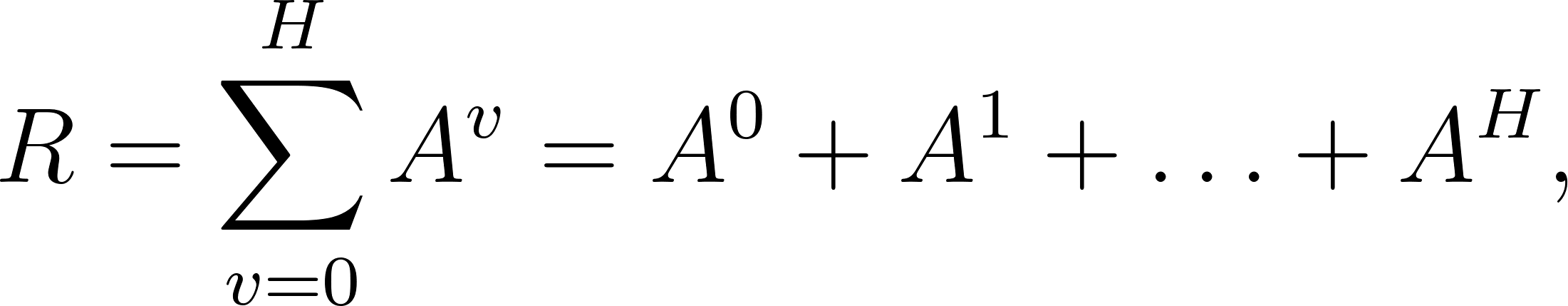
](https://www.codecogs.com/eqnedit.php?latex=%20R%20%3D%20%5Csum%5EH_%7Bv%3D0%7D%20A%5Ev%20%3D%20A%5E0%20%2B%20A%5E1%20%2B%20%5Cldots%20%2B%20A%5EH%2C%20#0)

where [
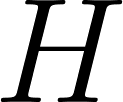
](https://www.codecogs.com/eqnedit.php?latex=H#0) is the height of the arbitrary tree. Each power [
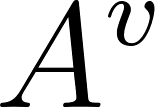
](https://www.codecogs.com/eqnedit.php?latex=A%5Ev#0) represents the nodes that can be reached from any node with a path of size [
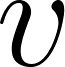
](https://www.codecogs.com/eqnedit.php?latex=v#0), so the matrix [
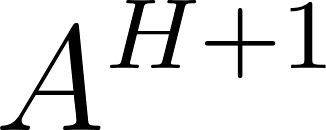
](https://www.codecogs.com/eqnedit.php?latex=A%5E%7BH%20%2B%201%7D#0) will be a zero matrix, as no two nodes have a distance that is larger than [
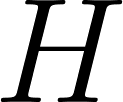
](https://www.codecogs.com/eqnedit.php?latex=H#0).

Another component is the sibling matrix [
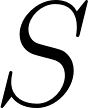
](https://www.codecogs.com/eqnedit.php?latex=S#0), which is also binary and encodes all local neighbors on the same level:

[
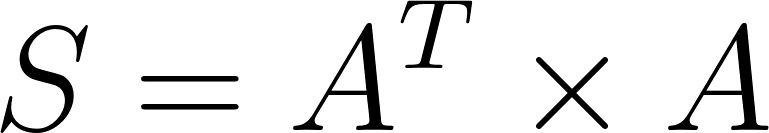
](https://www.codecogs.com/eqnedit.php?latex=%20S%20%3D%20A%5ET%20%5Ctimes%20A%20#0).

An example for all three matrices, [
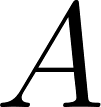
](https://www.codecogs.com/eqnedit.php?latex=A#0), [
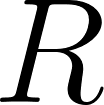
](https://www.codecogs.com/eqnedit.php?latex=R#0), and [
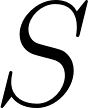
](https://www.codecogs.com/eqnedit.php?latex=S#0), based on the employed dataset and the associated label structure.

Using these matrices, the assignment of a voxel of the body to a specific class can be represented through conditional probabilities based on the tree structure. For instance, the probability of a voxel to belong to the right lower lobe of the lung depends on the probabilities of it belonging to the right lung, the lungs as a whole, the thoracic cavity, and the body. This concept is analogous to the one of Bayesian networks, which are acyclic directed graphs where each node contains probability information, and edges represent a direct influence between the nodes. In a Bayesian network, all relationships are expressed using conditional probabilities and can be simplified using the chain rule. This can also be applied to our hierarchical tree, but a normalization step needs to be added to ensure that each node represents a probability. This can be done using a softmax function, which is commonly employed as an activation function in neural networks for multi-class classification tasks. Its outputs are probabilities that indicate the likelihood of the input being assigned to each class, and its sum is 1, just like for probabilities.

These concepts can be used to build a deep learning model that uses conditional probabilities and the softmax function as activation layer for segmentation. The model takes an input and outputs a feature map [
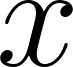
](https://www.codecogs.com/eqnedit.php?latex=x#0) with [
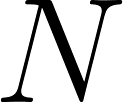
](https://www.codecogs.com/eqnedit.php?latex=N#0) channels, which is the number of nodes of the hierarchical tree. To enforce the hierarchical relationships, a final probability function for each class [
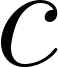
](https://www.codecogs.com/eqnedit.php?latex=c#0) can serve as the activation layer.

The probability [
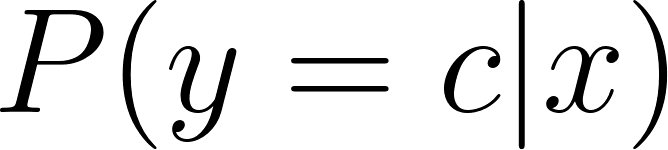
](https://www.codecogs.com/eqnedit.php?latex=P(y%20%3D%20c%20%7C%20x)#0) that the final class [
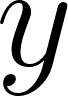
](https://www.codecogs.com/eqnedit.php?latex=y#0) corresponds to class [
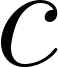
](https://www.codecogs.com/eqnedit.php?latex=c#0) can be computed using the chain rule as the product of the probabilities from the root node to node [
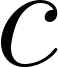
](https://www.zotero.org/google-docs/?JDM6FG). The probability of each node is normalized using the softmax for each sibling group, obtaining the following formula:

[
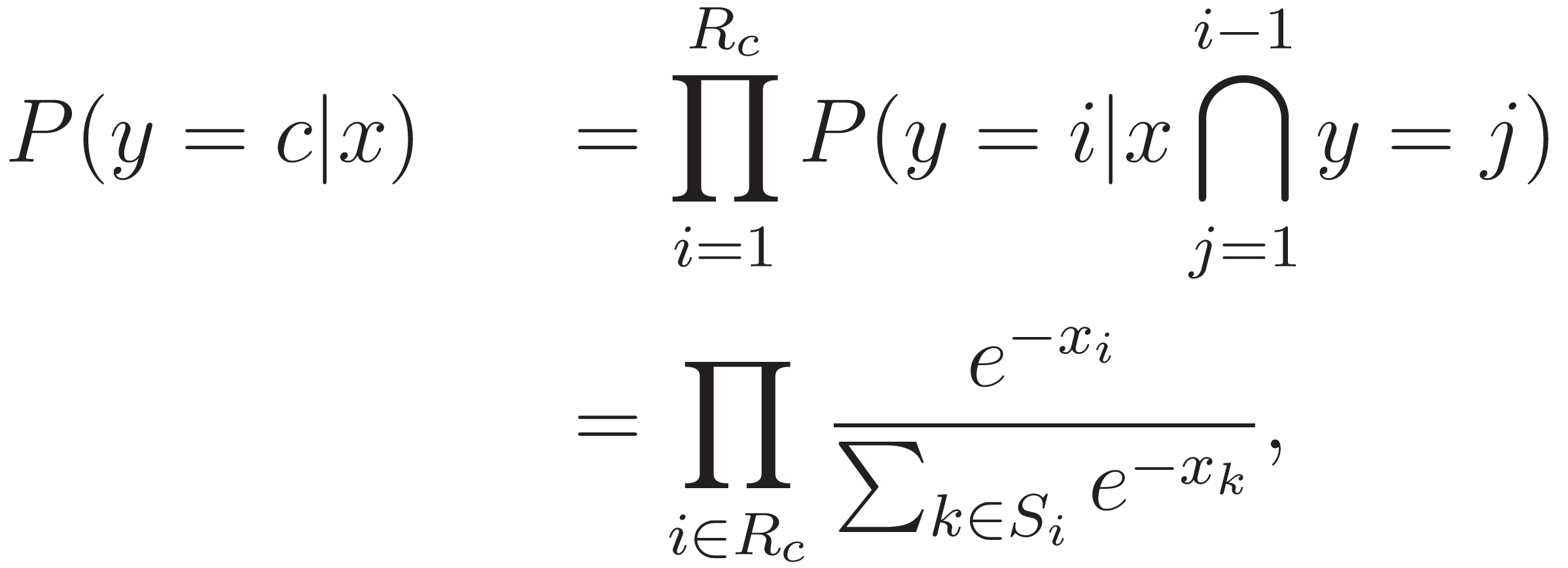
](http://www.sciweavers.org/tex2img.php?bc=Transparent&fc=Black&im=jpg&fs=100&ff=modern&edit=0&eq=P(y%3Dc%7Cx)%20%26%26%3D%20%5Cprod%5E%7BR_c%7D_%7Bi%3D1%7D%20P(y%3Di%7Cx%20%5Cbigcap%5E%7Bi-1%7D_%7Bj%3D1%7D%20y%20%3D%20j)%5C%5C%5C%5C%20%26%26%3D%5Cprod_%7Bi%20%5Cin%20R_c%7D%20%5Cfrac%7Be%5E%7B-x_i%7D%7D%7B%5Csum_%7Bk%20%5Cin%20S_i%7D%20e%5E%7B-x_k%7D%7D%2C#0)

Here, [
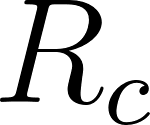
](https://www.codecogs.com/eqnedit.php?latex=R_c#0) denotes the column [
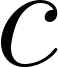
](https://www.codecogs.com/eqnedit.php?latex=c#0) of [
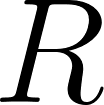
](https://www.codecogs.com/eqnedit.php?latex=R#0), which contains the nodes in the path between the root node and node [
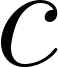
](https://www.codecogs.com/eqnedit.php?latex=c#0). [
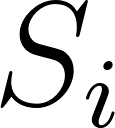
](https://www.codecogs.com/eqnedit.php?latex=S_i#0) represents the siblings of node , and  and  respectively correspond to the feature maps associated with node  and . The resulting probabilities of all leaf nodes sum up to one, analogously to the softmax function.

To illustrate an example of the probability chain, consider the process of determining the likelihood that a voxel is part of the left hip bone. This requires a sequential evaluation of the voxel's probability of being within the body, within the bone, then associated with hip bones, and finally identified as part of the specific left hip bone, which can be expressed as:

## 
